# *Atxn2* Knockout and CAG42-Knock-in Cerebellum Shows Similarly Dysregulated Expression in Calcium Homeostasis Pathway

**DOI:** 10.1007/s12311-016-0762-4

**Published:** 2016-02-11

**Authors:** Melanie Vanessa Halbach, Suzana Gispert, Tanja Stehning, Ewa Damrath, Michael Walter, Georg Auburger

**Affiliations:** 1 Experimental Neurology, Department of Neurology, Goethe University Medical School, Building 89, 3rd floor, Theodor Stern Kai 7, 60590 Frankfurt am Main, Germany; 2 Institute for Medical Genetics, Eberhard-Karls-University of Tuebingen, 72076 Tuebingen, Germany

**Keywords:** Atxn2, Itpr1, Rora, Calcium, Homeostasis, Cerebellum, Signaling

## Abstract

Spinocerebellar ataxia type 2 (SCA2) is an autosomal dominantly inherited neurodegenerative disorder with preferential affection of Purkinje neurons, which are known as integrators of calcium currents. The expansion of a polyglutamine (polyQ) domain in the RNA-binding protein ataxin-2 (ATXN2) is responsible for this disease, but the causal roles of deficient ATXN2 functions versus aggregation toxicity are still under debate. Here, we studied mouse mutants with *Atxn2* knockout (KO) regarding their cerebellar global transcriptome by microarray and RT-qPCR, in comparison with data from *Atxn2*-CAG42-knock-in (KIN) mouse cerebellum. Global expression downregulations involved lipid and growth signaling pathways in good agreement with previous data. As a novel effect, downregulations of key factors in calcium homeostasis pathways (the transcription factor *Rora*, transporters *Itpr1* and *Atp2a2*, as well as regulator *Inpp5a*) were observed in the KO cerebellum, and some of them also occurred subtly early in KIN cerebellum. The ITPR1 protein levels were depleted from soluble fractions of cerebellum in both mutants, but accumulated in its membrane-associated form only in the SCA2 model. Coimmunoprecipitation demonstrated no association of ITPR1 with Q42-expanded or with wild-type ATXN2. These findings provide evidence that the physiological functions and protein interactions of ATXN2 are relevant for calcium-mediated excitation of Purkinje cells as well as for ATXN2-triggered neurotoxicity. These insights may help to understand pathogenesis and tissue specificity in SCA2 and other polyQ ataxias like SCA1, where inositol regulation of calcium flux and RORalpha play a role.

**Electronic supplementary material:**

The online version of this article (doi:10.1007/s12311-016-0762-4) contains supplementary material, which is available to authorized users.

## Introduction

Spinocerebellar ataxias (SCAs) comprise a group of autosomal dominantly inherited disorders that are characterized by massive neurodegeneration preferentially in the cerebellum but also in other areas like spinal cord and brainstem [1]. The main shared symptom of these neurodegenerative processes is motor incoordination (gait, stance, and limb ataxia) [2, 3]. Additionally, there are non-motor symptoms that are characteristic for SCA subgroups like dementia or macular degeneration. Thus, the genetic basis and the initial phenotypes of diverse SCAs are heterogeneous, but their clinical and pathological features converge at later stages [4]. An important shared feature and a determinant of patients’ motor incoordination is the early vulnerability of cerebellar Purkinje neurons, which are known to function via integration of glutamatergic inputs like climbing fibers and parallel fibers [5]. Indeed, some emerging pathogenic pathways are shared among the SCAs and seem to involve the glutamate calcium-mediated excitation at the dendritic spines of cerebellar Purkinje neurons [6, 7]. However, for none of the SCAs, the preliminary insights into molecular pathogenesis have led to neuroprotective therapy.

The overall prevalence of SCAs is 5–7 cases per 100,000 people, with SCA2 being the second most prevalent variant [8]. SCA2 is caused by a CAG triplet repeat expansion in the *ATXN2* gene and, therefore, belongs to the group of CAG triplet repeat disorders like SCA1, SCA3, SCA6, SCA7, SCA17, Huntington’s Disease (HD), Dentatorubral pallidoluysian atrophy (DRPLA), and Spinal and bulbar muscle atrophy (SBMA) [9, 10]. In comparison to other SCAs, the SCA2 phenotype is characterized by saccadic slowing, hyporeflexia, myoclonus/fasciculations and postural/action tremor in early disease stages [11–13], Parkinsonian signs in later disease stages [14, 15], and prominent precerebellar/reticulotegmental/cranial nuclei/midbrain/thalamic/somatosensory/motoneuron degeneration apart from the classic olivo-ponto-cerebellar atrophy (OPCA) upon autopsy [16–21]. In 90 % of the human population, the *ATXN2* CAG repeat on chromosome 12q24 encodes a polyQ domain of about 22–23 glutamines encoded by the sequence (CAG)8-CAA-(CAG)4-CAA-(CAG)8or9 [22, 23]. Individuals with an expansion > Q32 have a high risk to develop SCA2. The ages of onset and of death are negatively correlated with the CAG repeat size [24]. Furthermore, intermediate length CAG repeats (27–31 units) often with remaining CAA interruptions have been shown to be a risk factor for amyotrophic lateral sclerosis (ALS), frontotemporal dementia (FTLD), progressive supranuclear palsy, and autosomal dominant Parkinson’s disease (PD) [25–31].

Physiologically, the ATXN2 protein shows abundant expression in many cell populations throughout the organism, such as cerebellar Purkinje neurons, hippocampal pyramidal neurons, or liver hepatocytes [32]. In cervical cancer HeLa cells, ATXN2 was identified as a protein with direct RNA binding [33]. It has been described as a cytoplasmic protein associating with the rough endoplasmic reticulum (ER) and colocalizing with ribosomes [34]. ATXN2 is involved in the regulation of global RNA processing and ribosomal translation by binding via its PAM2 motif to PABPC1 and via its LSM/LSMAD domains directly to RNA [35, 36]. Furthermore, a role in stress response exists, with ATXN2 relocalizing to the RNA quality control machinery in stress granules together with the seeding factor TIA-1 [37]. Finally, a small number of studies implicated ATXN2 also in trophic signaling, cytoskeletal reorganization, or nuclear transcription [38–41].

It is clear that polyglutamine (polyQ) diseases involve neurotoxicity due to a gain-of-function of the mutant proteins, but controversy exists about the pathogenic roles of fibril formation and aggregation as well as the contributions of a partial loss-of-function [42–45]. In SCA2 mouse models, the polyQ expansion of ATXN2 leads to sequestration of its physiological interaction partner poly(A)-binding protein C1 (PABPC1) into insolubility [46]. This should affect global mRNA turnover and protein synthesis in many neuron populations but cannot stand alone to explain the selective neurodegeneration pattern of SCA2. The genetic ablation of *Atxn2* in mice does not lead to a neurodegenerative process with weight loss such as SCA2, but instead to obesity, lipid anomalies, and insulin resistance [47, 48]. Given that ATXN2 binds to the poly(A)-tail of mRNAs, we decided to perform an unbiased global survey of the cerebellar transcriptome in *Atxn2* KO mice and then to test if the particularly strong expression dysregulations are mirrored in our previously characterized SCA2 mouse model with knock-in (KIN mice) of a CAG42 expansion into the *Atxn2* gene [46]. This approach allows us to discern loss-of-function and gain-of-function effects on mRNAs by ATXN2 mutations. We observed a prominent interesting dysregulation in calcium homeostasis pathways, validated them independently at the RNA and protein level, and assessed these effects in different brain regions and at various ages. Mechanistically, a progressive accumulation of ITPR1 in the relatively insoluble tissue fraction occurred selectively in the SCA2 mouse model, but the expected sequestration of this calcium release protein by endogenous amounts of Q42-ATXN2 was not detectable in coimmunoprecipitation and colocalization studies.

## Material and Methods

### Animals

Generation and characterization of *Atxn2* KO and *Atxn2*-CAG42-KIN mice has been described formerly [46, 47]. For KO mice, deletion of *Atxn2* exon 1 was achieved through Cre-Lox recombination and confirmed by quantitative real-time reverse transcriptase PCR (RT-qPCR) or by Western blot in the mice and all tissues under study. For KIN mice, the single CAG typical for the murine sequence of *Atxn2* exon 1 was expanded to 42 CAGs by homologous recombination. Mice were backcrossed from mixed 129/Ola (KO) and 129Sv/Pas × C57BL/6 (KIN) background into C57BL/6 for more than eight generations. Animals were housed in individually ventilated cages with fixed light cycle under routine health monitoring at the FELASA-certified Central Animal Facility (ZFE) of the Goethe University Medical School, Frankfurt am Main. They were fed ad libitum and bred in heterozygous matings. For dissection, mice were sacrificed by cervical dislocation. Subsequently, cerebella were removed and frozen immediately in liquid nitrogen. Tissue was stored at −80 °C until further use. All procedures were in accordance with the German Animal Welfare Act, the Council Directive of 24 November 1986 (86/609/EWG) with Annex II, and the ETS123 (European Convention for the Protection of Vertebrate Animals).

### Genotyping

Tail biopsies were used for genotyping and DNA was isolated using Proteinase K (Ambion) treatment and ethanol precipitation. For *Atxn2* KO PCR, 50 ng of DNA, 16 μl Pink Juice [125 μM Cresol Red Sodium Salt (Sigma Aldrich), 12.5 % 10× PCR buffer with 15 mM MgCl_2_ (Applied Biosystems), 250 μM dNTPs (Thermo Scientific), 25 % sucrose], 1 μl of the forward (*5′-TTG CCC CTT CTT GAG ACT GG-3′*) and each of the two reverse primers (*5′-GTA GAA CTG GGT GAT GGG GT-3′* and *5′-TGA GTA GCA AAA GCA AGG CC-3′*), as well as 0.1 μl Taq Polymerase (AmpliTaq^®^ DNA Polymerase, Applied Biosystems) were used. PCR conditions were as follows: initial denaturation at 95 °C for 3 min, 35 cycles of 94 °C for 30 s, 57 °C for 30 s, and 72 °C for 50 s, plus a final elongation step of 7 min at 72 °C. Predicted band sizes are 443 bp for the wild-type (WT) and 239 bp for the KO allele.

For *Atxn2*-CAG42-KIN PCR, 50 ng of DNA, 16.25-μl H_2_O, 2.5 μl 10× Buffer, 4 μl dNTPs (both Takara Bio Inc., Japan), 0.5 μl of forward (*5′-TGA GTT GAC TCC ACA GGG AGG TGA GC-3′*) and 0.5 μl of reverse (*5′-CCA TCT CGC CAG CCC GTA AGA TTC-3′*) primers, as well as 0.25 μl of LA Taq polymerase (Takara Bio Inc., Japan) were used. The following PCR conditions were applied: 3 min initial denaturation at 94 °C, followed by 30 cycles of 94 °C for 15 s, and 68 °C for 4 min, as well as a final elongation step of 9 min at 68 °C. The predicted length of the WT and KIN alleles are 793 and 984 bp, respectively.

### Global Transcriptome Profiling

After dissection, cerebellar tissue from 6-week- and 6-month-old *Atxn2* KO and WT animals (4 *Atxn2* ^+/+^ vs*.* 4 *Atxn2* ^−/−^ for each age) was sent to MFT Services (Tübingen, Germany). RNA was extracted and its quality was verified. Then, 100 ng of total RNA was amplified, labeled, and biotinylated with the GeneChip HT 3′IVT Express Kit (Affymetrix, Santa Clara, CA, USA). From the labeled and fragmented cRNA, 15 μg was hybridized with the GeneChip HT Mouse Genome 430 2.0 Array Plates (Affymetrix) and then washed, stained, and scanned automatically in a GeneTitan instrument (Affymetrix). The microarray chips can detect more than 39,000 transcripts covering 34,000 genes. Hybridization artifacts and proper grid alignment were controlled by visual inspection of the scanned images. Raw data were obtained with AGCC 3.0 software (Affymetrix) and stored in CEL files. The software platform R 2.14.0 and Bioconductor (www.bioconductor.org) were used for further analysis starting with background correction of the complete expression information and Robust Multichip Average (RMA) normalization. F-statistics was applied (empirical Bayes model) and the resulting *P* values were further corrected for multiple testing using the “Benjamini-Hochberg” method. After this correction, transcripts with *P* values <0.05 were considered as significantly dysregulated. The complete microarray transcriptome results were deposited in the public database GEO (http://www.ncbi.nlm.nih.gov/geo/query/acc.cgi?acc=GSE55177).

### Unbiased Transcriptome Bioinformatics

#### STRING database interaction analysis

The factors from Table 1 were loaded via the Multiple Names entry (http://string.embl.de/). Automatized visualization of connections between calcium regulators was kept. Remaining random factors were manually grouped by function.

**Table 1:** In *Atxn2* KO cerebellum at ages of 6, 12, 24 weeks, global transcriptome analysis showed mRNA levels of *Atxn2* and 32 non-anonymous genes downregulated with fold-changes below −1.31-fold (decreases beyond 0.66)

ID	Gene title	Gene symbol	F-test			Fold change		
			F	*P* value	adj. *P* value	6 weeks	12 weeks	24 weeks
1419866_s_at	Ataxin 2	Atxn2	174.48	2.12E − 39	3.18E − 35	−29.59	−21.59	−42.96
1460653_at	Ataxin 2	Atxn2	154.70	6.54E − 38	5.90E − 34	−9.99	−13.48	−14.01
1438143_s_at	Ataxin 2	Atxn2	153.71	7.86E − 38	5.91E − 34	−49.20	−21.51	−51.99
1438144_x_at	Ataxin 2	Atxn2	58.92	2.40E − 26	6.41E − 24	−2.09	−1.36	−4.20
1424034_at	RAR-related orphan receptor alpha	Rora	55.94	9.42E − 26	2.03E − 23	−1.36	−11.54	−1.32
1449001_at	Isovaleryl coenzyme A dehydrogenase	Ivd	28.18	2.62E − 18	1.09E − 16	−2.26	−8.36	−1.31
1459363_at	Ataxin 2	Atxn2	23.58	1.60E − 16	4.61E − 15	−1.80	−2.57	−1.46
1418427_at	Kinesin family member 5B	Kif5b	22.51	4.50E − 16	1.19E − 14	−1.95	−1.73	−1.56
1443516_at	Ataxin 2	Atxn2	22.13	6.60E − 16	1.68E − 14	−1.41	−4.29	−1.77
1460167_at	Aldehyde dehydrogenase family 7, member A1”	Aldh7a1	21.49	1.25E − 15	3.03E − 14	−1.94	−1.61	−1.44
1434553_at	Transmembrane protein 56	Tmem56	19.05	1.70E − 14	3.29E − 13	−1.60	−2.32	−1.42
1418429_at	Kinesin family member 5B	Kif5b	18.62	2.76E − 14	5.16E − 13	−2.18	−3.77	−1.77
1444128_at	Phosphatidylinositol-4-phosphate 5-kinase, type II, beta	Pip5k2b	17.13	1.58E − 13	2.60E − 12	−2.69	−7.19	−5.56
1435462_at	Phosphatidylinositol-specific phospholipase C, X domain containing 2	Plcxd2	17.01	1.82E − 13	2.96E − 12	−1.49	−4.45	−1.43
1452091_a_at	RNA binding motif protein 28	Rbm28	15.76	8.53E − 13	1.23E − 11	−1.71	−1.35	−1.50
1434832_at	Forkhead box O3a	Foxo3a	15.40	1.35E − 12	1.88E − 11	−1.48	−3.47	−1.41
1423978_at	SH3-binding kinase 1	Sbk1	14.95	2.43E − 12	3.26E − 11	−1.52	−8.07	−1.80
1433605_at	Inositol polyphosphate-5-phosphatase A	Inpp5a	14.60	3.85E − 12	5.01E − 11	−1.35	−2.88	−1.48
1426500_at	Isoprenylcysteine carboxyl methyltransferase	Icmt	14.23	6.36E − 12	7.96E − 11	−1.35	−3.73	−1.71
1460059_at	Uridine phosphorylase 2	Upp2	13.19	2.69E − 11	3.07E − 10	−1.33	−3.59	−1.44
1438408_at	Ankyrin repeat domain 56	Ankrd56	12.82	4.55E − 11	5.01E − 10	−1.45	−3.82	−1.57
1418468_at	Annexin A11	Anxa11	11.29	4.51E − 10	4.22E − 09	−1.31	−2.44	−1.40
1434831_a_at	Forkhead box O3a	Foxo3a	11.02	6.87E − 10	6.24E − 09	−1.33	−2.63	−1.37
1452426_x_at	Zinc finger protein 236	Zfp236	10.97	7.41E − 10	6.69E − 09	−2.35	−22.46	−3.14
1460670_at	RIO kinase 3 (yeast)	Riok3	10.61	1.31E − 09	1.14E − 08	−1.94	−2.32	−2.26
1434806_at	Metaxin 3	Mtx3	10.38	1.90E − 09	1.62E − 08	−1.40	−2.23	−1.45
1449254_at	Secreted phosphoprotein 1	Spp1	8.24	7.71E − 08	5.01E − 07	−2.09	−1.67	−1.44
1434932_at	Adenosine deaminase, RNA-specific, B1	Adarb1	8.24	7.71E − 08	5.01E − 07	−1.36	−1.45	−1.47
1446353_at	Tubulin, beta 6	Tubb6	7.56	2.75E − 07	1.63E − 06	−1.72	−1.81	−1.32
1448249_at	Glycerol-3-phosphate dehydrogenase 1 (soluble)	Gpd1	6.90	9.87E − 07	5.33E − 06	−1.33	−1.38	−1.68
1436876_at	Regulator of G-protein signalling 7 binding protein	Rgs7bp	6.08	5.10E − 06	2.43E − 05	−1.34	−1.54	−1.40
1448546_at	Ras association (RalGDS/AF-6) domain family 3	Rassf3	5.80	9.17E − 06	4.15E − 05	−1.45	−1.50	−1.32
1417010_at	Zinc finger protein 238	Zfp238	5.76	1.00E − 05	4.50E − 05	−1.90	−1.62	−1.75
1452714_at	Tetratricopeptide repeat, ankyrin repeat and coiled-coil containing 1	Tanc1	5.37	2.29E − 05	9.59E − 05	−1.32	−1.80	−1.37
1457412_at	Sodium channel, voltage-gated, type VIII, alpha	Scn8a	4.96	5.56E − 05	2.14E − 04	−1.35	−1.41	−1.46
1424007_at	Growth differentiation factor 10	Gdf10	4.48	1.63E − 04	5.68E − 04	−2.10	−2.97	−1.73
1422847_a_at	Protein kinase C, delta	Prkcd	4.26	2.72E − 04	8.98E − 04	−2.38	−2.27	−1.83
1417279_at	Inositol 1,4,5-triphosphate receptor 1	Itpr1	3.12	3.87E − 03	9.45E − 03	−1.55	−1.75	−1.70
1416551_at	ATPase, Ca++ transporting, cardiac muscle, slow twitch 2	Atp2a2	68.07	5.11E − 28	2.28E − 25	−1.20	−4.19	−1.16
1452363_a_at	ATPase, Ca++ transporting, cardiac muscle, slow twitch 2	Atp2a2	19.94	6.41E − 15	1.35E − 13	−1.21	−2.30	−1.23

The genes are listed in order of significance by adjusted *P* values (4 *Atxn2* ^+/+^ vs*.* 4 *Atxn2* ^−/−^for each age)

#### Gene Set Enrichment Analysis

Gene symbols and M-values of microarray data from 12-week-old cerebellum were subjected to nonspecific filtering, and the remaining genes were analyzed using Gene Set Enrichment Analysis (GSEA) and the Java-based version GSEA-P [49, 50]. For each comparison, the probe IDs were ranked according to the *t* test statistic. Probe IDs were collapsed to gene symbols. For duplicate entries, the maximum value was used. Permutations were performed on gene sets due to the low number of biological replicates. We used the c2 (online pathway databases, PubMed publications, expert of domain knowledge) genesets from the MSigDB database (v4.0, May 2013, http://www.broadinstitute.org/gsea/msigdb/index.jsp) to analyze the data sets.

### RNA Isolation and Expression Analysis

For expression analysis, RNA was extracted from cerebellar tissue (25 mg) of the relevant mice with TRIzol^®^ reagent (Invitrogen) according to the manufacturers’ protocol. Remaining DNA was digested with DNase I Amplification Grade (Invitrogen) before cDNA synthesis. Reverse transcription was performed with SuperScript III Reverse Transcriptase (Invitrogen) and expression levels were measured by RT-qPCR with the StepOnePlus Real-Time PCR System (Applied Biosystems). Therefore, cDNA from 25-ng RNA, 10 μl of FastStart Universal Probe Master (Rox) Mix (Roche), and 1 μl of one of the following TaqMan Assays (Applied Biosystems) were used for each reaction: *Atp2a2* (Mm01201431_m1), *Inpp5a* (Mm00805812_m1), *Itpr1* (Mm00439907_m1), *Rora* (Mm01173766_m1), as well as *Tbp* (Mm00446973_m1) and *Hprt1* (Mm00446968_m1) as endogenous controls. PCR conditions were 50 °C for 2 min, followed by 10 min at 95 °C and 40 cycles of 95 °C for 15 s and 60 °C for 60 s. Gene expression data was analyzed using the 2^−ΔΔCt^ method [51]. Additionally, mean Ct values from *Tbp* and *Hprt1* were averaged to avoid false positive results in one of the housekeeping genes. Then, Ct values from the respective transcript were normalized to *Tbp + Hprt* and to the average of the WT values.

### Protein Extraction and Quantitative Immunoblotting

For SDS polyacrylamide gel electrophoresis (PAGE), immunoblotting, and quantitative densitometry, protein was extracted from 25 mg cerebellar tissue of either 6-month-old *Atxn2* KO and WT or 18-month-old *Atxn2*-CAG42-KIN and WT mice. The tissue was homogenized in 10 vol. RIPA buffer (50 mM Tris–HCl (pH 8.0); 150 mM NaCl; 1 mM EDTA; 1 mM EGTA; 1 % Igepal CA-630 (Sigma); 0.5 % sodium deoxycholate; 0.1 % SDS; 1 mM PMSF; Complete Protease Inhibitor Cocktail (Roche)) with a motor pestle and incubated on ice for 15 min. Samples were then centrifuged for 20 min at 4 °C and 16,000×*g* and the supernatant was transferred into a new tube and kept on ice until further processing (RIPA fraction). Using sonification, the remaining pellet was dissolved in ½ vol. 2× SDS buffer (137 mM Tris–HCl (pH 6.8); 4 % SDS; 20 % glycerol; Complete Protease Inhibitor Cocktail (Roche)), centrifuged for 10 min at 16,000×*g* and the remaining supernatant was separated as SDS fraction. For protein concentration determination, the BCA protein assay kit (Interchim, France) was applied and the received values were normalized to the respective buffers. Samples were boiled with 2× loading buffer (25 % stacking gel buffer (0.5 M Tris, 0.4 % SDS; pH 6.8), 20 % Glycerol, 4 % SDS, 5 % β-Mercaptoethanol, and 0.05 % Bromophenol blue) at 95 °C for 5 min to denature proteins. Subsequently, 20 μg of each protein lysate was loaded onto a 7 % polyacrylamide gel and after electrophoresis transferred to a nitrocellulose membrane. Blocking was performed in 5 % slim-milk powder in PBST for 1 h. Primary antibodies were used at the following dilutions: INPP5A (MyBiosource, MBS716862, 1:1000) and ITPR1 (Abcam, ab5804, 1:1000 and Millipore ABS55, 1:1000). Fluorescently tagged secondary antibodies were used (LI-COR Odyssey Infrared Imaging, goat anti-mouse IRDye 800CW and goat anti-rabbit IRDye 680RD, both 1:15,000), and proteins were detected using a LI-COR Odyssey Infrared Imaging System. Densitometry was performed with ImageJ software and protein values were normalized to β-ACTIN levels using EXCEL.

### Coimmunoprecipitation

For coimmunoprecipitation (Co-IP) studies, cerebellum from 8-week-old *Atxn2*-CAG42-KIN and *Atxn2* KO and WT mice was homogenized in 1:10 w/v NP40 buffer (20 mM Tris–HCl pH 8.0; 137 mM NaCl; 1 % Glycerol; 0.1 % Igepal CA-630 (Sigma); 2 mM EDTA; Complete Protease Inhibitor Cocktail (Roche)) using a motor pestle and incubated for 15 min on ice. Subsequently, samples were centrifuged for 20 min at 16,000×*g* and 4 °C and supernatant was transferred into a new tube. Prior to the immunoprecipitation step, 20 μl of Protein A agarose beads (Santa Cruz) were washed twice with lysis buffer (120 mM NaCl; 0.1 % Triton X 100; 50 mM Tris–HCl pH 7.5) for 5 min. A centrifugation step of 1 min at 2300×*g* followed before the supernatant was discarded. To decrease the adhesiveness of the beads, they were incubated for 1 h at room temperature (RT) on a rotating wheel together with 1 ml of blocking buffer (0.2 % NaCl; 0.1 % gelatin; 0.05 % NaN_3_; 50 mM Tris; 0.1 % Triton). At the same time, 250 μg of cerebellar protein extract was preincubated with the respective pulling antibodies against ATXN2 (50 μl/sample, custom-made) or ITPR1 (10 μl/sample, Abcam ab5804) for 2 h at 4 °C on a rotating wheel. Beads were then centrifuged for 1 min at 2300×*g* and blocking buffer was removed before the preincubated protein extract was added. Subsequently, samples were incubated at 4 °C on a rotating wheel overnight and centrifuged for 1 min at 2300×*g*. The supernatant was removed and samples were washed three times with 1 ml of lysis buffer and centrifuged again for 1 min at 2300×*g*. After discarding the supernatant, samples were boiled for 5 min at 95 °C with 25 μl loading buffer and loaded onto the 8 % SDS gel. The immunoblot detection of ATXN2 was carried out with the commercial rabbit polyclonal IgG antibody from ProteinTech (catalog no. 21776-1-AP) at titer 1:500, employing the IRdye680 donkey anti-rabbit (1:15,000 at RT for 1 h) as secondary antibody.

### Immunohistochemical Staining

Paraffin-embedded slices were used after rehydration in a descending ethanol series. Between incubation steps, slices were stored or washed in Tris/HCl buffer pH 7.6. For antigen retrieval, slices were autoclaved (Biocare Medical) in Bull’s Eye Decloaker (1:20). The following conditions were applied: 125 °C for 30 s and 90 °C for 10 s. Slides were subsequently cooled down and washed. Slices were incubated in 100 % methanol, 30 % H_2_O_2_, and Tris/HCl pH 7.6 (1:1:8) for 30 min in a wet chamber for background reduction. After another washing step, they were blocked in 2.5 μl Triton-X-100, 18.2 mg DL-Lysine, and 998 μl 5 % Tris–BSA for 30 min. Then, the slices were incubated with the first antibodies (anti-ATXN2, BD Biosciences, 611378, 1:50 and anti-ITPR1, Abcam, ab5804, 1:800) overnight. After another washing step, incubation with the secondary fluorescently labeled antibodies followed for 6 h (Cy3 and Cy2, Dianova, 711-225-152 and 715-165-150, 1:1000). Finally, slices were mounted with DAKO fluorescent mounting medium. Microscopic pictures were taken with a Nikon confocal microscope Eclipse 90i and a ×60 magnification.

### Statistical Analysis

Data analysis was conducted with GraphPad Prism software version 4.03 (2005) using Student’s *t* test. Error bars indicate SEM. Significant *P* values (<0.05) were marked as follows: *p* < 0.05*, *p* < 0.01**, *p* < 0.001***. A trend (T) was noted when 0.05 < *P* < 0.1.

## Results

### Microarray Transcriptome Profile of *Atxn2* KO Cerebellum Shows Dysregulation of Several Factors Involved in Calcium Homeostasis Pathways

Attempting to gain deeper insight into the function of the RNA-binding protein ATXN2 and the cerebellar consequences of its deficiency, we performed global transcriptome analysis of *Atxn2* KO mice. Cerebella from 6-week-, 3-month-, and 6-month-old animals, in each case four wild-type (WT, *Atxn2* ^+/+^) versus four homozygous knockout (KO, *Atxn2* ^−/−^) mice, were examined for mRNA level changes using a total of 24 Affymetrix oligonucleotide microarray chips. The data confirmed the absence of *Atxn2* mRNA at six spots for all three ages with changes ranging from −51.99-fold to −1.36-fold (Table 1), with variance probably due to technical imprecisions in independent assessments. Apart from *Atxn2*, the analysis of significant downregulations with consistency across ages revealed a list of 126 transcript coding for 109 genes. Table 1 shows the 32 non-anonymous genes with changes stronger than −1.31-fold (corresponding to a decrease to 0.66, or log2-fold-changes/M-values ≤ −0.39) among them, ordered by significance of the adjusted *P* value. Interaction analysis at the String Heidelberg database highlighted the calcium homeostasis pathway to be affected (Supp. Fig. 1), but multiple factors of RNA processing (*Riok3, Rbm28, Adarb1*), bioenergetics (*Aldh7a1, Gpd1*), cell adhesion (*Tanc1, Tmem56*), growth (*Icmt, Gdf10, Foxo3, Rassf3, Sbk1*), and lipid signaling (*Pip4k2b, Plcxd2, Rgs7bp*) were also present in this short list. Further bioinformatics assessment of the complete transcriptome of 12-week-old cerebella by Gene Set Enrichment Analysis (GSEA) at the Broad Institute server also revealed significant downregulations in the KEGG adherens junction pathway of cell adhesion, the BIOCARTA PPARA pathway of lipid signaling, the BIOCARTA EGF pathway of growth signaling, and the BIOCARTA Creb pathway of nuclear response to extracellular triggers with *Camk2b* (calcium/calmodulin-dependent protein kinase II beta) as the strongest downregulation (Suppl. Fig. 2–5). These results are in good agreement with previous observations of phenotypic effects in the lipid metabolism, insulin, and EGF signaling [38, 39, 47]. The most significant change after *Atxn2* was observed for *Rora* as a transcription factor that regulates several genes involved in calcium homeostasis besides having other effects (Table 1), *Inpp5a* and *Itpr1* as two regulators of calcium flux and signaling besides other functions, as well as *Prkcd* and *Anxa11* as calcium-regulated targets were present among the top 32 genes, and age-consistent significant downregulations (−1.2-fold) were observed at two individual microarray spots for the calcium flux regulator *Atp2a2* (Table 1, bottom), as a possible correlate of altered calcium signal transmission at Purkinje neuron dendrites. Thus, this unbiased analysis of cerebellar transcriptome dysregulations with consistency through three ages advanced previous knowledge on altered RNA processing/bioenergetics/cell adhesion/growth/lipid signaling and documented novel prominent downregulations of mRNAs for proteins involved in calcium homeostasis and signaling that are caused by *Atxn2* loss.

### Downregulation of Factors Involved in Calcium Homeostasis Pathways is Confirmed Independently by qPCR

To validate the results of the transcriptome analysis and to investigate even younger ages as well as other brain regions in *Atxn2* KO homozygous and heterozygous animals, an approach by RT-qPCR was used. The downregulation of *Atp2a2*, *Inpp5a*, *Itpr1*, and *Rora* mRNA levels in cerebellum of 6-month-old *Atxn2* ^−/−^animals was thus confirmed by an independent technique (Table 2; *n* ≥ 4 *Atxn2* ^+/+^ vs*.* ≥3 *Atxn2* ^−/−^). Fold changes were similar to those found by the microarray profiling, apart from *Itpr1* that showed a much smaller downregulation.

**Table 2:** *Atp2a2*, *Inpp5a*, *Itpr1*, and *Rora* were confirmed to be downregulated in *Atxn2* KO mice using RT-qPCR

Gene symbol	WT vs*.* HOM KO Cbll 6 months
*Atp2a2*	−1.13-fold*
*Inpp5a*	−1.30-fold**
*Itpr1*	−1.05-fold*
*Rora*	−1.42-fold**
Gene symbol	WT *vs.* HOM KO Cbll 6 weeks
*Atp2a2*	−1.24-fold*
*Inpp5a*	−1.39-fold**
*Itpr1*	−1.05-fold n.s.
*Rora*	−1.11-fold**
Gene symbol	WT vs*.* HET KO Cbll 6 weeks
*Atp2a2*	−1.11-fold*
*Inpp5a*	−1.35-fold**
*Itpr1*	−1.07-fold n.s.
*Rora*	−1.15-fold***
Gene symbol	WT *vs.* HET KO Cbll 3 days
*Atp2a2*	−1.17-fold**
*Inpp5a*	−1.19-fold**
*Itpr1*	+1.04-fold n.s.
*Rora*	−1.14-fold T

Downregulation of the transcripts was detected by qPCR in cerebellum. *Atp2a2* and *Inpp5a* showed the first but *Rora* the strongest downregulation. Significant changes are marked with asterisks while trends are indicated with “T” (*n* ≥ 4 *Atxn2* ^+/+^ vs*.* n ≥ 3 *Atxn2* ^−/−^)

*T* statistical trend with *P* value between 0.05 and 0.10, *n.s*. non-significant, *Cbll* Cerebellum

*P < 0.05; **P < 0.01; ***P < 0.001

To test the temporal dynamics of these effects, 6-week-old *Atxn2* ^−/−^ mice were studied (*n* ≥ 4 *Atxn2* ^+/+^ vs*.* ≥4 *Atxn2* ^−/−^). To further analyze the effects independently in animals with reduced mutation load, heterozygous (*Atxn2* ^+/−^) mice at the age of 6 weeks (*n* ≥ 4 *Atxn2* ^+/+^ vs*.* 4 *Atxn2* ^+/−^) were analyzed. Finally, in order to test the earliest appearance of these effects in animals with reduced mutation load, heterozygous pups at ~3 days of age (*n* = 4 *Atxn2* ^+/+^ vs*.* 4 *Atxn2* ^+/−^) were assessed. As shown in Table 2, consistent results for all these conditions were observed, with significant downregulations of similar strength for *Atp2a2* and *Inpp5a*, as well as downregulations of weaker strength for *Rora*. These observations indicate that changes in calcium homeostasis pathways are present very early even in heterozygous *Atxn2* KO cerebellum.

### *Atxn2*-CAG42-KIN Mice Show Subtle Transcript Level Changes in Calcium Homeostasis Pathways

Aware that RORA has been shown to be an important modifier of the cerebellar ataxia in SCA1 mouse models [52], we extended the analysis to the cerebellum of SCA2 mouse models. The *Atxn2*-CAG42-KIN (*Atxn2* ^CAG42/CAG42^) mouse cerebellum at an age of 18 months was assessed by RT-qPCR. At this age, the *Atxn2*-CAG42-KIN mice show the first behavioral deficits and visible aggregates of the ATXN2 protein in cerebellar Purkinje neurons [46]. In contrast to KO mice at this age, only *Atp2a2* and *Itpr1* were slightly but significantly downregulated in homozygous *Atxn2*-CAG42 KIN mice while *Inpp5a* and *Rora* did not show any change at all (*n* ≥ 4 *Atxn2* ^+/+^ vs*.* ≥4 *Atxn2* ^CAG42/CAG42^) (Table 3).

**Table 3:** Subtle downregulation of transcripts involved in calcium homeostasis pathways in *Atxn2*-CAG42-KIN mice

Gene symbol	WT vs*.* HOM KIN Cbll 18 months
*Atp2a2*	−1.17-fold**
*Inpp5a*	+1.00-fold n.s.
*Itpr1*	−1.15-fold*
*Rora*	−1.01-fold n.s.
Gene symbol	WT vs*.* HOM KIN Cbll 6 months
*Atp2a2*	−1.12-fold*
*Inpp5a*	−1.05-fold n.s.
*Itpr1*	−1.12-fold**
*Rora*	+1.00-fold n.s.
Gene symbol	WT vs*.* HOM KIN Cbll 6 weeks
*Atp2a2*	−1.05-fold n.s.
*Inpp5a*	−1.05-fold n.s.
*Itpr1*	−1.12-fold*
*Rora*	−1.07-fold n.s.

*Atp2a2* and *Itpr1* were significantly downregulated, with *Itpr1* showing the earliest effect in cerebellum. Significant changes are marked with asterisks while trends are indicated with “T” (*n* ≥ 4 *Atxn2* ^+/+^ vs*.* n ≥ 3 *Atxn2* ^*C*AG42/CAG42^)

*T* statistical trend with *P* value between 0.05 and 0.10, *n.s*. non-significant, *Cbll* cerebellum

*P < 0.05; **P < 0.01; ***P < 0.001

To again document the temporal dynamics, the transcription analysis was extended to the cerebellum of 6-month- and 6-week-old *Atxn2*-CAG42-KIN mice. While *Atp2a2* and *Itpr1* were significantly downregulated (*n* ≥ 4 *Atxn2* ^+/+^ vs*.* ≥3 *Atxn2* ^CAG42/CAG42^) at 6 months of age with similar strength (about -1.15-fold) as at 18 months of age, only *Itpr1* was downregulated at 6 weeks of age (*n* ≥ 4 *Atxn2* ^+/+^ vs*.* ≥4 *Atxn2* ^CAG42/CAG42^) (-1.12-fold). These data indicate an unexpected preferential and early affection of *Itpr1* mRNA levels in the SCA2 model cerebellum, which is not observed in the *Atxn2* KO tissue.

### Dysregulated Protein Levels in Calcium Homeostasis Pathways in *Atxn2* KO and *Atxn2*-CAG42-KIN Mice

**Fig. 1:**
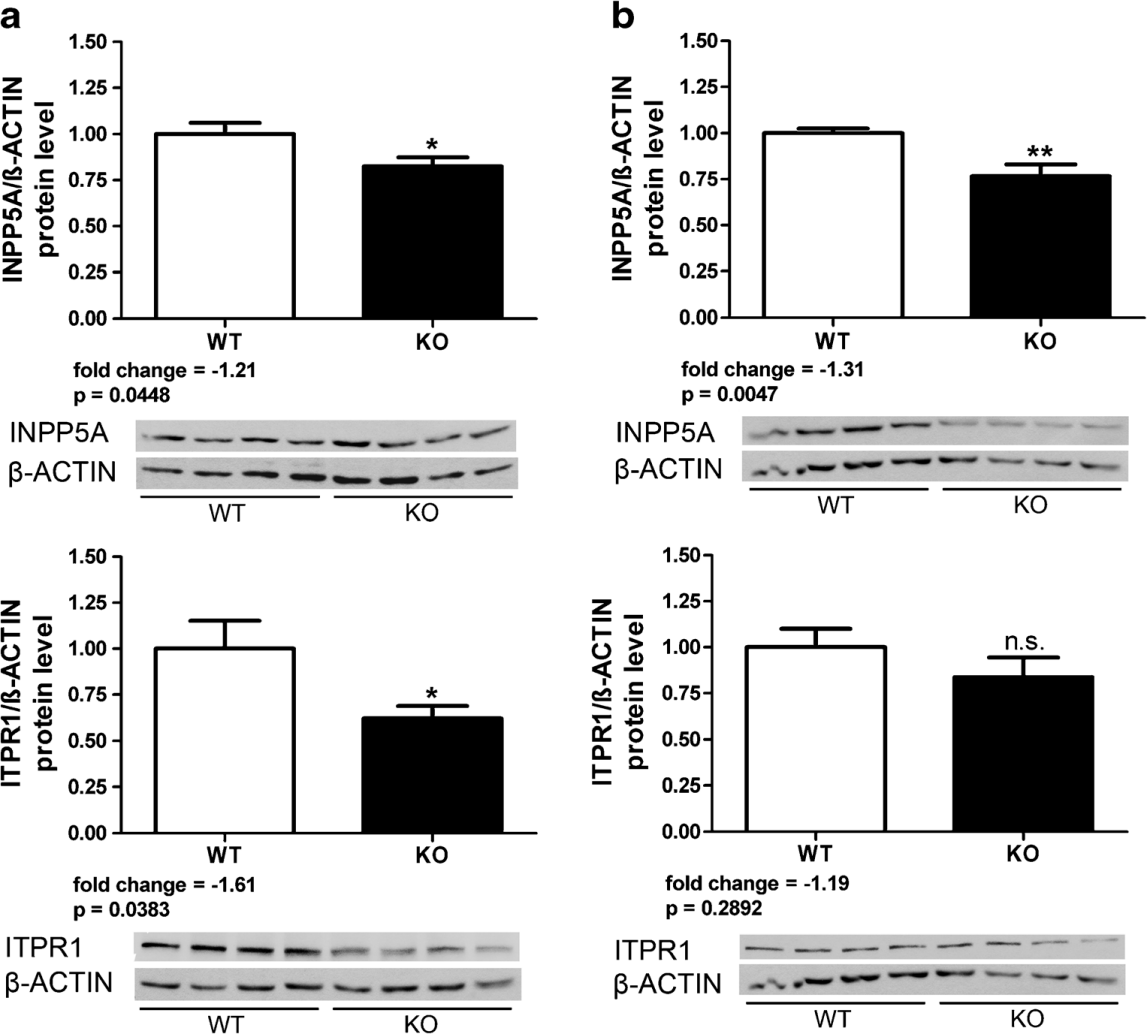
Downregulation of INPP5A and ITPR1 protein levels in *Atxn2* KO mouse cerebellum. Protein levels were measured in RIPA-soluble (**a**) and SDS-soluble (**b**) fraction of cerebellar tissue from 6-month-old animals (8 *Atxn2* ^+/+^ vs*.* ≥7 *Atxn2* ^−/−^). Both proteins were downregulated in the RIPA fraction, but only INPP5A was also downregulated in the SDS fraction

Despite the modest fold changes of the expression dysregulations observed, we attempted to validate the corresponding protein levels. In view of the limited availability of specific antibodies, only INPP5A and ITPR1 levels were assessed via quantitative immunoblots in cerebellar tissue from 6-month-old mice (8 *Atxn2* ^+/+^ vs*.* ≥7 *Atxn2* ^−/−^). In the RIPA-soluble fraction (Fig. 1a) where mostly cytosolic factors will be detected, both proteins were significantly downregulated (fold changes −1.21-fold for INPP5A and −1.61-fold for ITPR1). In the SDS-soluble fraction (Fig. 1b), where membrane-associated proteins such as cortical actin or also aggregating proteins may be solubilized, only INPP5A showed significant downregulation (−1.31-fold). These data demonstrate that the relatively subtle transcript dysregulations are indeed resulting in substantially altered abundance of the corresponding proteins, making an impairment of calcium flux regulation in *Atxn2* KO mouse cerebellum more credible.

**Fig. 2:**
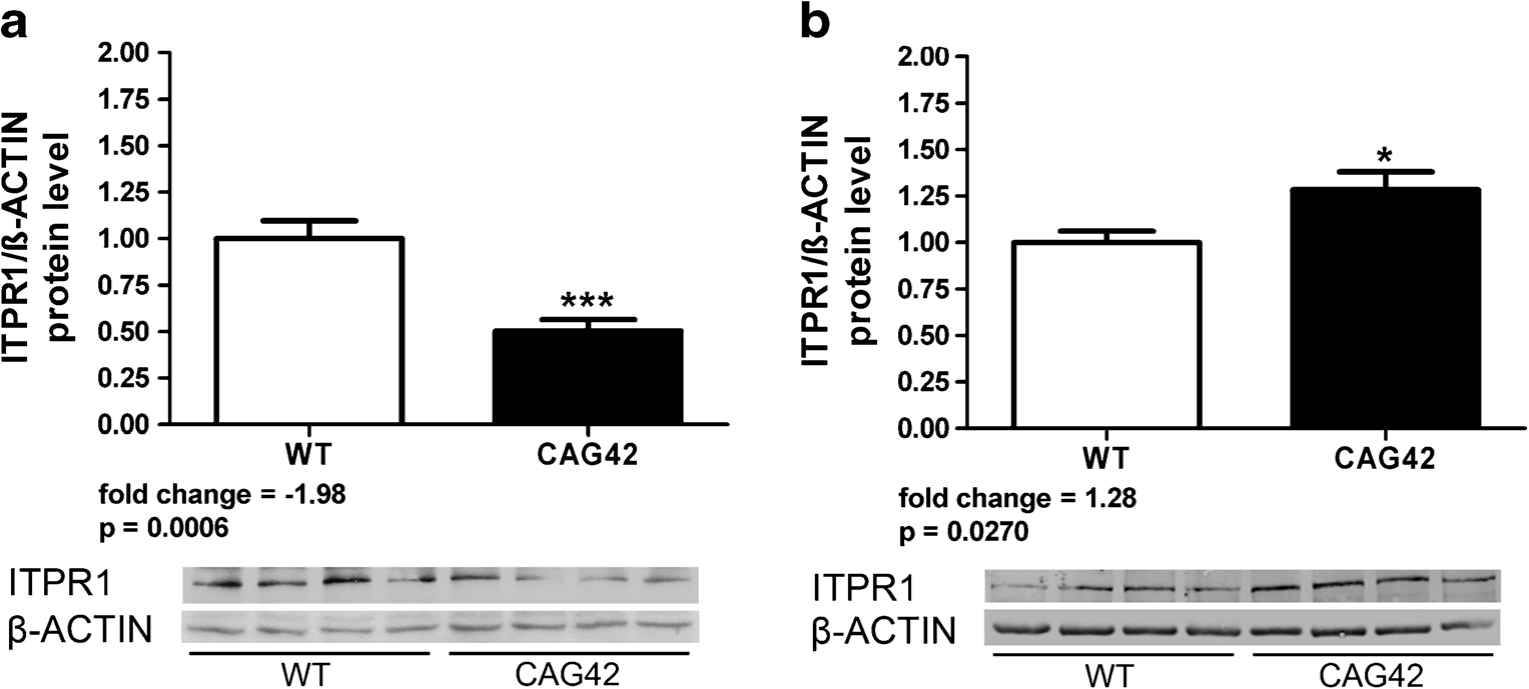
ITPR1 protein shifted into insolubility in *Atxn2*-CAG42-KIN cerebellum. Protein levels from cerebellar tissue of 18-month-old WT and KIN animals (8 *Atxn2* ^+/+^ vs*.* 7 *Atxn2* ^CAG42/CAG42^) were analyzed in the RIPA-soluble (**a**) and SDS-soluble (**b**) fraction. ITPR1 was decreased in the RIPA fraction, while it accumulated in SDS fraction

In the KIN mouse line, we focused on ITPR1 only, since the *Inpp5a* mRNA levels were not dysregulated. Protein lysates from cerebellar tissue (18-month-old animals) were studied with quantitative immunoblots (8 *Atxn2* ^+/+^ vs*.* 7 *Atxn2* ^CAG42/CAG42^), demonstrating significantly diminished (−1.98-fold) ITPR1 protein levels in the RIPA-soluble fraction of *Atxn2*-CAG42-KIN mice (Fig. 2a). Conversely, significantly increased (1.28-fold) ITPR1 protein levels were observed in the SDS-soluble fraction of *Atxn2-*CAG42-KIN mice (Fig. 2b). In view of the previously demonstrated aggregation of ATXN2 protein in the SDS/urea fraction [46] and reports on a protein interaction between ATXN2 and ITPR1 [53, 54], this accumulation of ITPR1 in relative insolubility with concomitant depletion from the soluble tissue fraction might reflect a sequestration of ITPR1 by polyQ-expanded ATXN2.

### Normal and 42Q-ATXN2 do not Interact with ITPR1 at Endogenous Protein Levels

**Fig. 3:**
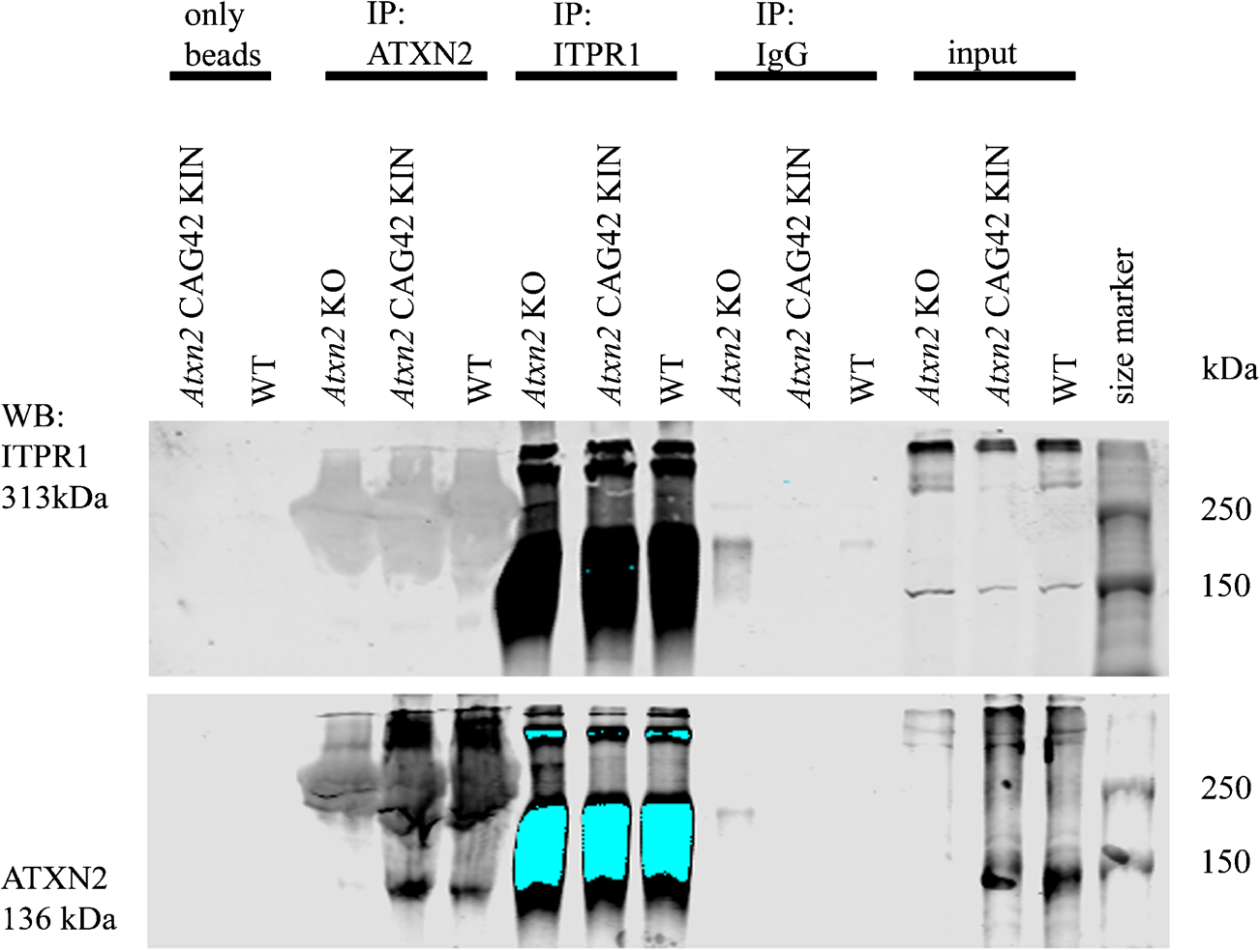
No interaction of ITPR1 with normal and expanded ATXN2 in *Atxn2*-CAG42-KIN cerebellum. Co-IPs pulling either with beads only, ATXN2, ITPR1, or unspecific IgG control managed to precipitate both ITPR1 (*panel above*) and ATXN2 (*panel below*) at endogenous levels, but showed no interaction of ITPR1 with wildtype or Q42-expanded ATXN2 in immunoblots detecting ITPR1

Given that a protein interaction between ITPR1 and overexpressed 58Q-expanded ATXN2 was previously observed [54], we went on to study both endogenous proteins in their association via Co-IP in the cerebellum of *Atxn2*-CAG42-KIN and WT mice at age 18 months, using *Atxn2* KO tissue as negative control. Pulling was done with anti-ATXN2 on the one hand or with anti-ITPR1 on the other hand, performing pull-downs with unspecific IgG or using beads only as negative controls. ITPR1 (Fig. 3, panel above) and ATXN2 (panel below) at endogenous levels were readily detectable as input in tissues of all genotypes and were concentrated by its immunoprecipitation, providing the necessary quality control. However, ITPR1 bands were not brought down in detectable amounts by WT or Q42-ATXN2. These results suggest that ATXN2 polyQ expansion sizes such as Q42, which are found in most SCA2 patients, are unable at endogenous levels within the first years of pathology progression to mediate a strong sequestration of ITPR1.

### ITPR1 Localization in Purkinje Cells is not Visibly Altered due to ATXN2 Expansion or Loss

**Fig. 4:**
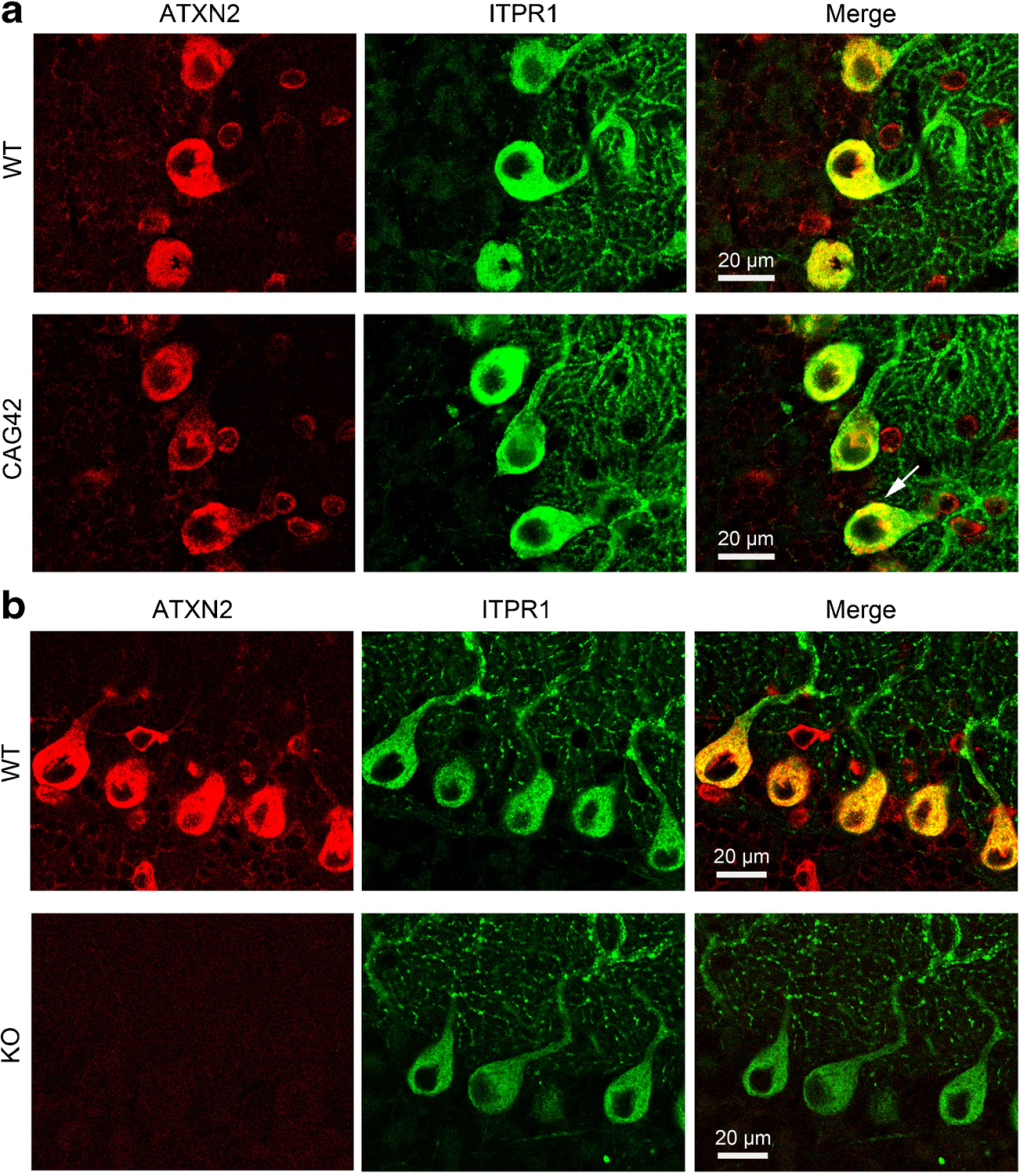
Colocalization of ITPR1 and ATXN2. Fluorescent double immunohistochemical stainings of ATXN2 (*red*) and ITPR1 (*green*) in **a** 24-month-old *Atxn2*-CAG42-KIN and **b** 6-month-old *Atxn2* KO cerebellum. ATXN2 and ITPR1 colocalized in the cytoplasm of Purkinje cells. While ITPR1 staining intensity varied considerably among individual Purkinje neurons of the same cerebellum, the ITPR1 localization was not detectably influenced by ATXN2 polyQ expansion or loss

In order to study the basis of the ITPR1 accumulation in relative insolubility among membrane-soluble factors in *Atxn2*-CAG42-KIN mice and of the ITPR1 abundance reduction in *Atxn2* KO mice, immunohistochemical double stainings of ATXN2 and ITPR1 were performed in both mouse lines. Several sections from sagittal brain slices of 24-month-old WT and *Atxn2*-CAG42-KIN as well as of 6-month-old WT and *Atxn2* KO mice (two animals per line) were applied and stained for ATXN2 (red) and ITPR1 (green) (Fig. 4a, b). Qualitative assessment without sophisticated quantitative techniques was carried out. ATXN2 showed cytoplasmic distribution in Purkinje cells of both WT mice. In *Atxn2*-CAG42-KIN Purkinje neurons, ATXN2 was aggregated (arrow), as previously reported [46]. As expected, no specific fluorescent signal was detected for ATXN2 in *Atxn2* KO cerebellum. ITPR1 exhibited a granular distribution in the cytoplasm as well as in the dendrites of Purkinje cells. Colocalization of ATXN2 and ITPR1 was therefore detected throughout the cytoplasm in WT cells, but the ITPR1 localization appeared largely unchanged when ATXN2 was expanded or lost. No obvious accumulation, relocalization, or markedly decreased intensity in the staining was apparent in the KIN or KO. Furthermore, no change in Purkinje cell soma size was detectable neither in *Atxn2*-CAG42-KIN nor in *Atxn2* KO cerebellum. Thus, although less salt-soluble ITPR1 and more detergent-soluble ITPR1 was observed in *Atxn2*-CAG42-KIN mice, a sequestration of ITPR1 to aggregates or inclusion bodies was not detected, suggesting that the ITPR1 accumulation occurs in its normal membrane-associated form.

## Discussion

Our global transcriptome survey of *Atxn2* KO cerebellum defined significant downregulations of key molecules in several pathways where previous studies had demonstrated phenotypic changes, namely extracellular growth/adhesion signals via lipid and transcription changes. As a prominent novel effect with selectivity for cerebellum, the mRNA levels of calcium homeostasis pathway components *Atp2a2*, *Inpp5a*, *Itpr1*, and particularly *Rora* showed varying decreases. This insight may provide parameters to measure how the loss of physiological function of ATXN2 affects the integration of calcium signals in the dendritic trees of Purkinje dendrites. It is also useful to understand how the physiological function of ATXN2 is altered by polyQ expansions in SCA2. Thus, our data for the first time show clearly significant dysregulations in the abundance of several factors involved in calcium homeostasis pathways, in the cerebellum of a Q42-knock-in mouse model of SCA2, but these changes are mild even at old age and are probably not the sole basis for the marked pathology already manifest. These factors define a pathway of Purkinje neuron signaling. Climbing fiber and parallel fiber afferents excite Purkinje cell dendrites via glutamate receptors, resulting in the hydrolysis of phosphatidylinositol bisphosphate (PIP_2_) into diacylglycerol (DG) and inositol 1,4,5-trisphosphate (InsP_3_). InsP_3_ then binds ITPR1 which thereupon releases Ca^2+^ from the endoplasmic reticulum into the cytosol. The stimulator InsP_3_ itself can be hydrolyzed by inositol polyphosphate-5-phosphatase (INPP5A) producing InsP_2_ that is unable to activate the InsP_3_ receptor. This mechanism inhibits further stimulation of calcium release [53]. The SERCA2 protein, encoded by *Atp2a2*, counteracts ITPR1 by translocating Ca^2+^ back into the endoplasmic reticulum lumen [55]. *Rora* is of specific interest as a transcription factor that regulates several genes involved in calcium homeostasis and signaling like *Calb1* (encoding Calbindin), *Grm1* (encoding the metabotropic glutamate receptor, which influences calcium signaling through excitatory synaptic neurotransmission), and *Itpr1* [56], among other effects. Overall, calcium-mediated excitation influences the duration, direction, extent, and type of synaptic plasticity, thus underlying the pathomechanism of diverse neurological diseases [57].

The calcium signaling pathway has been implicated in the pathogenesis of several spinocerebellar ataxias. *Inositol 1,4,5-trisphosphate receptor 1* (ITPR1) is mutated in SCA15 and was implicated early on also in other human and murine SCAs [58–60]. A downregulation of *Itpr1* and *Inpp5a* expression at the transcript level was already reported for several other SCA mouse models: Sca1[82Q] transgenic mice [52, 61], Sca1^−/−^ and Sca1^154Q/+^ mice [62], as well as Sca3[Q79] transgenic (only *Itpr1*) [63] and Sca7^266Q/+^ mice (only *Inpp5a*) [64]. A downregulation of *Atp2a2* was shown in Sca1[82Q] transgenic [52, 61], Sca1^−/−^ and Sca1^154Q/+^ [62] mouse models but also in a mouse mutant called staggerer [52, 56]. The staggerer mice have a spontaneous mutation of the *Rora* gene, resulting in complete loss of RORA function and congenital ataxia. Similar to the genes mentioned above, *Rora* was shown to be dysregulated in Sca1[Q82] transgenic [52] and Sca3[69Q] transgenic mice [65]. Additionally, an increase in Rora level (by Tip60 loss) can delay cerebellar degeneration in the Sca1[Q82] transgenic mouse model [66]. Thus, these alterations appear to be common to various SCA variants as part of a downstream pathway of cerebellar pathogenesis and may be useful as molecular read-outs of presymptomatic or clinically manifest ataxia.

It is interesting to meditate about the varying mechanisms how this calcium pathway can be affected by different SCA disease proteins. An interaction with the transcription factor RORA was demonstrated for the nuclear factor Ataxin-1 [52]. In contrast, Ataxin-2 is a cytoplasmic protein with concentration at the rough endoplasmic reticulum. There, its overexpressed 58Q-expanded variant was observed to bind to ITPR1 [7, 54, 67]. Although these studies found the overexpression of INPP5A by adeno-associated virus over 17 weeks and until 10 months to alleviate the Purkinje neuron phenotypes and motor incoordination in mice with stable transgenic overexpression of a human CAG58-*ATXN2* cDNA, our data in the GAG42-knock-in mouse model of SCA2 show no significant upregulation of the *Inpp5a* transcript, but instead consistent downregulations of the *Atp2a2* mRNA to occur at ages before and after the manifestation of motor incoordination. It is impossible to predict the net effect of these changes in abundance of antagonistic calcium modulators without future functional studies of electrophysiology and calcium imaging in knock-in mice. The mild changes at the RNA level translate into substantial changes at the protein level: INPP5A protein is downregulated in the KO cerebellum. Furthermore, ITPR1 is markedly altered in its levels and solubility both by ATXN2-Q42 expansion and by ATXN2 deficiency. Given that a sequestration of ITPR1 into insolubility through protein interaction with expanded ATXN2 could not be substantiated in our coimmunoprecipitation and colocalization studies, the likeliest explanation would predict that the reduced amounts of *Itpr1* mRNA result in reduced amounts of newly synthesized ITRP1 at ribosomes, soluble in the RIPA fraction, but the cells accumulate ITPR1 in its ER membrane-associated form, which is represented by the SDS fraction. This might be a compensatory stabilization effort of the cells to maintain calcium homeostasis. The previous observation of protein interactions between ITPR1 and ATXN2 by Liu and coworkers [54] may be explained by the longer polyQ expansion in ATXN2, by its overexpression, and by different experimental conditions with a more stringent lysis buffer.

An alternative pathomechanism instead of protein interaction is also conceivable. Expanded ATXN2 could associate with the *Itpr1* mRNA and influence its translation efficiency. Recently, it was demonstrated that ATXN2 is involved in the stability of mRNA and protein expression by binding to AU-rich sequences in the 3′UTR [68]. These authors furthermore found that a depletion of ATXN2 resulted in decreased mRNA stability and protein levels of specific targets. A polyQ expansion of ATXN2 did not abolish its function in this process but decreased its efficiency. In our analysis, the downregulations of *Atp2a2*, *Inpp5a*, *Itpr1*, and *Rora* mRNA levels in microarray transcriptomics and in RT-qPCR was specific for cerebellum in KO mice and was robustly detectable from 3 days to 6 months of age, even upon partial loss-of-function. *Atp2a2* and *Inpp5a* transcript level changes were the first to be detected in KO cerebellum, even in heterozygous tissue. Conversely, in KIN cerebellum, the downregulation of *Itpr1* levels was the most pronounced and earliest detectable change, while *Atp2a2* was significantly downregulated from age 6 months onward. Thus, in *Atxn2* KO mice, the loss of ATXN2 may result in decreased transcript and protein levels due to a decreased mRNA stability, while in *Atxn2*-CAG42-KIN mice, the mRNA stability may be partially decreased, compensatory efforts may stabilize the protein through modulation of its degradation speed, and additional pathology may occur in later disease stages or in stronger expansions due to ATXN2 accumulation/aggregation/sequestration.

The accumulation of ITPR1 in the membrane fraction in association with polyQ-expanded ATXN2 would affect Ca^2+^ release, a feature that could be tested in young mice but is cumbersome to evaluate in aged brain tissues. This effort seems hard to justify, given that SCA2 involves not only the cerebellum but progresses into a multisystem-atrophy of the nervous system, and that our data suggest the calcium homeostasis pathway alteration not to be prominent in tissues like midbrain and motoneurons (data not shown). It will be particularly important to determine if the effects of ATXN2 polyQ expansions on the motor neuron degeneration in amyotrophic lateral sclerosis has shared pathogenesis pathways.

Our present findings clarify the position of ATXN2 effects in the emerging shared molecular pathways of spinocerebellar ataxias. Previous data had suggested a role of ATXN2 in SCA1, given that double mutant *Drosophila melanogaster* flies showed increased dAtx2 levels to enhance Ataxin-1[82Q]-induced neurodegeneration, while decreased dAtx2 levels suppress the neurotoxicity [69]. Furthermore, ATXN2 appears to have also a role in SCA3, given that dAtx2 activity hastens the onset of nuclear inclusions associated with SCA3 [70]. The dependence of ITPR1, INPP5A, ATP2A2, and RORA levels on ATXN2 mutations provides proof-of-principle of the existence of shared molecular changes early in the course of SCA2, SCA1, and SCA3.

Further assessment of additional Atxn2-KO mouse tissues by global deep proteomics substantiated the downregulation of iso-valeryl dehydrogenase (IVD), which had shown the second strongest transcript downregulation in this microarray transcriptomics analysis. The IVD protein reduction had nominal significance in cerebellum and showed also actual significance after multiple testing correction in liver tissue, where a decrease to 7 % of WT levels was observed in quantitative immunoblots. This effect was part of a systematic downregulation of amino acids and fatty acids pathways, which is thought to affect excitation and growth signals [71]. Given that deep proteomics is not sensitive toward quantitative changes of factors with very low abundance like transcription factors, and that tissue extractions even with 8 % SDS may solubilize nuclear proteins only partially, it is not surprising that RORalpha changes were not detected by this approach.

## Electronic supplementary material

Below is the link to the electronic supplementary material.

Supplemental Figure 1 STRING interaction analysis of factors with 1.3-fold mRNA level downregulation in cerebellum of *Atxn2*-KO mice. The String database server Heidelberg was used to input multiple names of the factors in Table 1 and illustrate the archived experimental and text mining data on protein interactions. The central role of calcium homeostasis pathway factors was automatically recognized. Recent additional literature knowledge on functional similarities between the factors was used to manually rearrange the order into groups of RNA processing, bioenergetics, cell adhesion, growth and lipid signaling. (JPG 77 kb)

Supplemental Figure 2 GSEA summary on KEGG adherens junction downregulations. (PDF 85 kb)

Supplemental Figure 3 GSEA summary on BIOCARTA PPARA pathway downregulation. (PDF 74 kb)

Supplemental Figure 4 GSEA summary on BIOCARTA EGF pathway downregulation. (PDF 68 kb)

Supplemental Figure 5 GSEA summary on BIOCARTA CREB pathway downregulation. (PDF 65 kb)
