# Supplementary figures and images for: Atxn2 Knockout and CAG42-Knock-in Cerebellum Shows Similarly Dysregulated Expression in Calcium Homeostasis Pathway
Source: Cerebellum. 2016 Feb 11;16(1):68–81. doi: 10.1007/s12311-016-0762-4 (PMC5243904; doi:10.1007/s12311-016-0762-4)

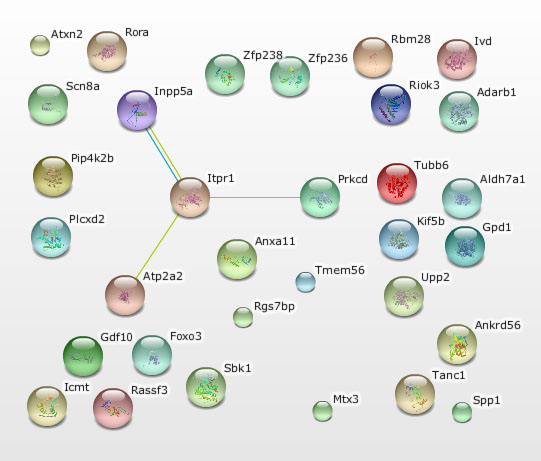

Supplement: Supplementary file 1 — STRING interaction analysis of factors with 1.3-fold mRNA level downregulation in cerebellum of Atxn2-KO mice. The String database server Heidelberg was used to input multiple names of the factors in Table 1 and illustrate the archived experimental and text mining data on protein interactions. The central role of calcium homeostasis pathway factors was automatically recognized. Recent additional literature knowledge on functional similarities between the factors was used to manually rearrange the order into groups of RNA processing, bioenergetics, cell adhesion, growth and lipid signaling. (JPG 77 kb) [file 12311_2016_762_MOESM1_ESM.jpg]
