# Supplementary material for: Atxn2 Knockout and CAG42-Knock-in Cerebellum Shows Similarly Dysregulated Expression in Calcium Homeostasis Pathway
Source: Cerebellum. 2016 Feb 11;16(1):68–81. doi: 10.1007/s12311-016-0762-4 (PMC5243904; doi:10.1007/s12311-016-0762-4)
Supplement: Supplementary file 2 — GSEA summary on KEGG adherens junction downregulations. (PDF 85 kb) [file 12311_2016_762_MOESM2_ESM.pdf]

Table: GSEA Results Summary

Dataset	GSEA_cbll_KO_collapsed
Phenotype	NoPhenotypeAvailable
Upregulated in class	na_neg
GeneSet	KEGG_ADHERENS_JUNCTION
Enrichment Score (ES)	-0.67744637
Normalized Enrichment Score (NES)	-1.8561758
Nominal p-value	0.0
FDR q-value	0.0017294717
FWER p-Value	0.002

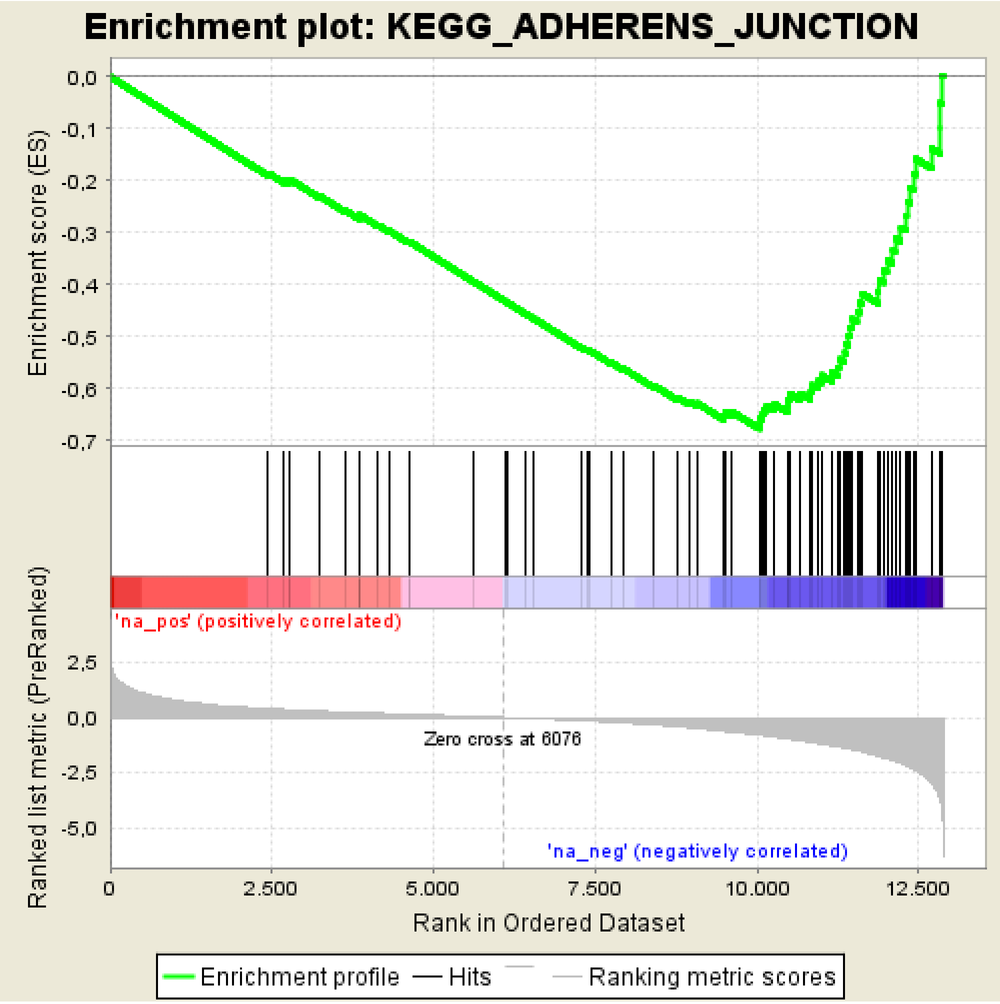

Fig 1: Enrichment plot: KEGG_ADHERENS_JUNCTION
Profile of the Running ES Score & Positions of GeneSet Members on the Rank Ordered List

Table: GSEA details [plain text format]

	PROBE	GENE	GENE_TITLE	RANK	RANK	RUNNING	CORE

		SYMBOL		IN GENE LIST	METRIC SCORE	ES	ENRICHMENT
1	CDC42	CDC42 Entrez, Source	cell division cycle 42 (GTP binding protein, 25kDa)	2438	0.398	-0.1851	No
2	PTPRM	PTPRM Entrez, Source	protein tyrosine phosphatase, receptor type, M	2689	0.360	-0.1999	No
3	RHOA	RHOA Entrez, Source	ras homolog gene family, member A	2757	0.350	-0.2005	No
4	ACTN2	ACTN2 Entrez, Source	actinin, alpha 2	2772	0.348	-0.1970	No
5	SNAI1	SNAI1 Entrez, Source	snail homolog 1 (Drosophila)	3246	0.286	-0.2302	No
6	ERBB2	ERBB2 Entrez, Source	v-erb-b2 erythroblastic leukemia viral oncogene homolog 2, neuro/glioblastoma derived oncogene homolog (avian)	3623	0.242	-0.2564	No
7	LEF1	LEF1 Entrez, Source	lymphoid enhancer-binding factor 1	3850	0.220	-0.2711	No
8	ACP1	ACP1 Entrez, Source	acid phosphatase 1, soluble	3852	0.220	-0.2683	No
9	CTNNA2	CTNNA2 Entrez, Source	catenin (cadherin-associated protein), alpha 2	3853	0.219	-0.2655	No
10	PTPN6	PTPN6 Entrez, Source	protein tyrosine phosphatase, non-receptor type 6	4141	0.190	-0.2854	No
11	TJP1	TJP1 Entrez, Source	tight junction protein 1 (zona occludens 1)	4307	0.172	-0.2960	No
12	WASF3	WASF3 Entrez, Source	WAS protein family, member 3	4611	0.144	-0.3178	No
13	WAS	WAS Entrez, Source	Wiskott-Aldrich syndrome (eczema-thrombocytopenia)	4612	0.144	-0.3159	No
14	PVRL4	PVRL4 Entrez, Source	poliovirus receptor-related 4	5616	0.045	-0.3936	No
15	SMAD2	SMAD2 Entrez, Source	SMAD, mothers against DPP homolog 2 (Drosophila)	6120	-0.003	-0.4328	No

16	SNAI2	SNAI2 Entrez, Source	snail homolog 2 (Drosophila)	6139	-0.005	-0.4341	No
17	MAP3K7	MAP3K7 Entrez, Source	mitogen-activated protein kinase kinase kinase 7	6432	-0.036	-0.4565	No
18	RAC3	RAC3 Entrez, Source	ras-related C3 botulinum toxin substrate 3 (rho family, small GTP binding protein Rac3)	6558	-0.050	-0.4656	No
19	IGF1R	IGF1R Entrez, Source	insulin-like growth factor 1 receptor	7289	-0.138	-0.5207	No
20	IQGAP1	IQGAP1 Entrez, Source	IQ motif containing GTPase activating protein 1	7372	-0.150	-0.5251	No
21	INSR	INSR Entrez, Source	insulin receptor	7424	-0.156	-0.5271	No
22	TCF7	TCF7 Entrez, Source	transcription factor 7 (T-cell specific, HMG-box)	7746	-0.208	-0.5494	No
23	ACTN3	ACTN3 Entrez, Source	actinin, alpha 3	7933	-0.237	-0.5608	No
24	CTNNA3	CTNNA3 Entrez, Source	catenin (cadherin-associated protein), alpha 3	8399	-0.327	-0.5928	No
25	ACTG1	ACTG1 Entrez, Source	actin, gamma 1	8786	-0.417	-0.6175	No
26	CDH1	CDH1 Entrez, Source	cadherin 1, type 1, E-cadherin (epithelial)	8950	-0.458	-0.6242	No
27	WASF1	WASF1 Entrez, Source	WAS protein family, member 1	9073	-0.497	-0.6272	No
28	FYN	FYN Entrez, Source	FYN oncogene related to SRC, FGR, YES	9498	-0.627	-0.6521	No
29	SRC	SRC Entrez, Source	v-src sarcoma (Schmidt-Ruppin A-2) viral oncogene homolog (avian)	9521	-0.636	-0.6455	No
30	SMAD3	SMAD3 Entrez, Source	SMAD, mothers against DPP homolog 3 (Drosophila)	9615	-0.663	-0.6440	No
31	NLK	NLK Entrez, Source	nemo-like kinase	10044	-0.795	-0.6670	Yes
32	TGFBR1	TGFBR1 Entrez, Source	transforming growth factor, beta receptor I (activin A receptor type II-like kinase,	10052	-0.796	-0.6571	Yes

			53kDa)				
33	CSNK2B	CSNK2B Entrez, Source	casein kinase 2, beta polypeptide	10084	-0.810	-0.6489	Yes
34	CTNNB1	CTNNB1 Entrez, Source	catenin (cadherin-associated protein), beta 1, 88kDa	10103	-0.817	-0.6396	Yes
35	LMO7	LMO7 Entrez, Source	LIM domain 7	10147	-0.836	-0.6320	Yes
36	MAPK1	MAPK1 Entrez, Source	mitogen-activated protein kinase 1	10270	-0.885	-0.6300	Yes
37	YES1	YES1 Entrez, Source	v-yes-1 Yamaguchi sarcoma viral oncogene homolog 1	10469	-0.973	-0.6327	Yes
38	BAIAP2	BAIAP2 Entrez, Source	BAI1-associated protein 2	10478	-0.977	-0.6205	Yes
39	PVRL3	PVRL3 Entrez, Source	poliovirus receptor-related 3	10501	-0.987	-0.6093	Yes
40	MAPK3	MAPK3 Entrez, Source	mitogen-activated protein kinase 3	10669	-1.047	-0.6086	Yes
41	TGFBR2	TGFBR2 Entrez, Source	transforming growth factor, beta receptor II (70/80kDa)	10829	-1.119	-0.6063	Yes
42	EP300	EP300 Entrez, Source	E1A binding protein p300	10843	-1.126	-0.5926	Yes
43	RAC2	RAC2 Entrez, Source	ras-related C3 botulinum toxin substrate 2 (rho family, small GTP binding protein Rac2)	10940	-1.169	-0.5847	Yes
44	FGFR1	FGFR1 Entrez, Source	fibroblast growth factor receptor 1 (fms-related tyrosine kinase 2, Pfeiffer syndrome)	10997	-1.200	-0.5734	Yes
45	PTPRB	PTPRB Entrez, Source	protein tyrosine phosphatase, receptor type, B	11157	-1.284	-0.5690	Yes
46	SSX2IP	SSX2IP Entrez, Source	synovial sarcoma, X breakpoint 2 interacting protein	11244	-1.333	-0.5582	Yes
47	CSNK2A1	CSNK2A1 Entrez, Source	casein kinase 2, alpha 1 polypeptide	11271	-1.344	-0.5426	Yes
48	SMAD4	SMAD4 Entrez, Source	SMAD, mothers against DPP homolog 4 (Drosophila)	11357	-1.385	-0.5311	Yes

49	MLLT4	MLLT4 Entrez, Source	myeloid/lymphoid or mixed-lineage leukemia (trithorax homolog, Drosophila); translocated to, 4	11373	-1.395	-0.5140	Yes
50	PTPRF	PTPRF Entrez, Source	protein tyrosine phosphatase, receptor type, F	11414	-1.414	-0.4986	Yes
51	VCL	VCL Entrez, Source	vinculin	11435	-1.427	-0.4814	Yes
52	CSNK2A2	CSNK2A2 Entrez, Source	casein kinase 2, alpha prime polypeptide	11475	-1.451	-0.4654	Yes
53	PARD3	PARD3 Entrez, Source	par-3 partitioning defective 3 homolog (C. elegans)	11547	-1.496	-0.4514	Yes
54	RAC1	RAC1 Entrez, Source	ras-related C3 botulinum toxin substrate 1 (rho family, small GTP binding protein Rac1)	11587	-1.523	-0.4345	Yes
55	CTNNA1	CTNNA1 Entrez, Source	catenin (cadherin-associated protein), alpha 1, 102kDa	11631	-1.565	-0.4173	Yes
56	ACTB	ACTB Entrez, Source	actin, beta	11873	-1.743	-0.4133	Yes
57	PVRL1	PVRL1 Entrez, Source	poliovirus receptor-related 1 (herpesvirus entry mediator C; nectin)	11886	-1.753	-0.3912	Yes
58	PTPN1	PTPN1 Entrez, Source	protein tyrosine phosphatase, non-receptor type 1	11957	-1.821	-0.3728	Yes
59	WASF2	WASF2 Entrez, Source	WAS protein family, member 2	12013	-1.871	-0.3526	Yes
60	FARP2	FARP2 Entrez, Source	FERM, RhoGEF and pleckstrin domain protein 2	12098	-1.956	-0.3335	Yes
61	PTPRJ	PTPRJ Entrez, Source	protein tyrosine phosphatase, receptor type, J	12137	-1.997	-0.3103	Yes
62	CTNND1	CTNND1 Entrez, Source	catenin (cadherin-associated protein), delta 1	12223	-2.082	-0.2896	Yes
63	ACTN1	ACTN1 Entrez, Source	actinin, alpha 1	12311	-2.192	-0.2677	Yes
64	TCF7L2	TCF7L2 Entrez, Source	transcription factor 7-like 2 (T-cell specific, HMG-box)	12320	-2.199	-0.2395	Yes
65	MET	MET Entrez,	met proto-oncogene (hepatocyte growth factor	12370	-2.280	-0.2134	Yes

		Source	receptor)				
66	SORBS1	SORBS1 Entrez, Source	sorbin and SH3 domain containing 1	12420	-2.354	-0.1864	Yes
67	ACTN4	ACTN4 Entrez, Source	actinin, alpha 4	12455	-2.401	-0.1576	Yes
68	WASL	WASL Entrez, Source	Wiskott-Aldrich syndrome-like	12702	-2.981	-0.1377	Yes
69	PVRL2	PVRL2 Entrez, Source	poliovirus receptor-related 2 (herpesvirus entry mediator B)	12833	-3.727	-0.0990	Yes
70	EGFR	EGFR Entrez, Source	epidermal growth factor receptor (erythroblastic leukemia viral (v-erb-b) oncogene homolog, avian)	12835	-3.735	-0.0501	Yes
71	CREBBP	CREBBP Entrez, Source	CREB binding protein (Rubinstein-Taybi syndrome)	12861	-4.097	0.0016	Yes

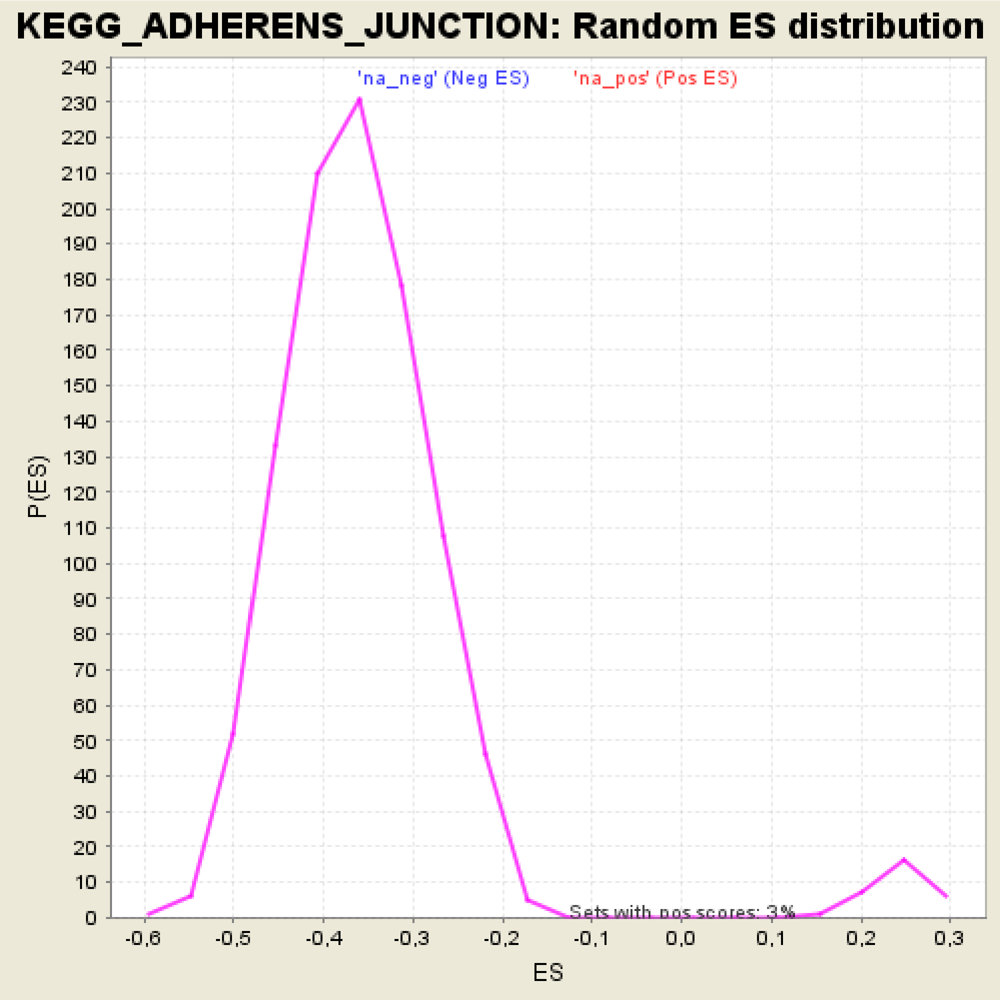

Fig 2: KEGG_ADHERENS_JUNCTION: Random ES distribution
Gene set null distribution of ES for KEGG_ADHERENS_JUNCTION
