# Supplementary material for: Atxn2 Knockout and CAG42-Knock-in Cerebellum Shows Similarly Dysregulated Expression in Calcium Homeostasis Pathway
Source: Cerebellum. 2016 Feb 11;16(1):68–81. doi: 10.1007/s12311-016-0762-4 (PMC5243904; doi:10.1007/s12311-016-0762-4)
Supplement: Supplementary file 3 — GSEA summary on BIOCARTA PPARA pathway downregulation. (PDF 74 kb) [file 12311_2016_762_MOESM3_ESM.pdf]

Table: GSEA Results Summary

Dataset	GSEA_cbll_KO_collapsed
Phenotype	NoPhenotypeAvailable
Upregulated in class	na_neg
GeneSet	BIOCARTA_PPARA_PATHWAY
Enrichment Score (ES)	-0.65223783
Normalized Enrichment Score (NES)	-1.7479517
Nominal p-value	0.0
FDR q-value	0.008572222
FWER p-Value	0.06

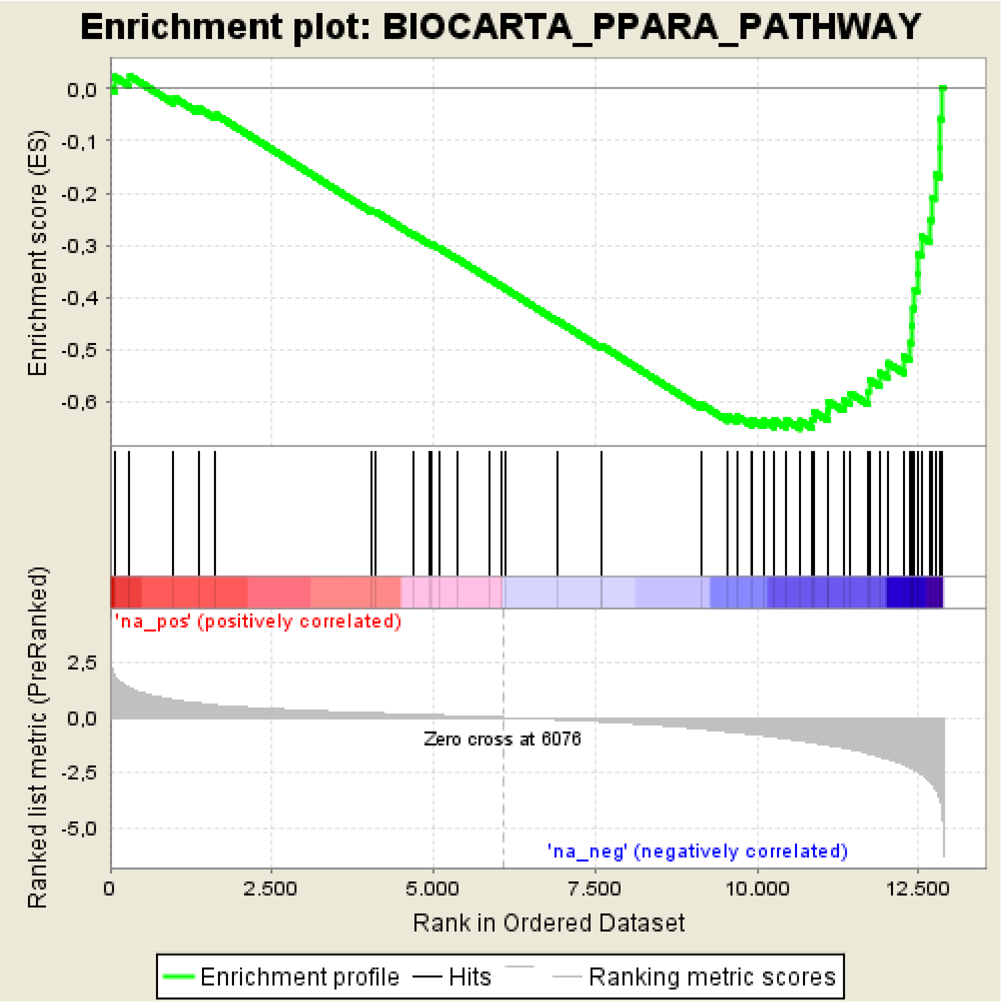

Fig 1: Enrichment plot: BIOCARTA_PPARA_PATHWAY
Profile of the Running ES Score & Positions of GeneSet Members on the Rank Ordered List

Table: GSEA details [plain text format]

	PROBE	GENE	GENE_TITLE	RANK	RANK	RUNNING	CORE

		SYMBOL		IN GENE LIST	METRIC SCORE	ES	ENRICHMENT
1	LPL	LPL Entrez, Source	lipoprotein lipase	71	1.902	0.0228	No
2	NR1H3	NR1H3 Entrez, Source	nuclear receptor subfamily 1, group H, member 3	305	1.330	0.0245	No
3	CD36	CD36 Entrez, Source	CD36 molecule (thrombospondin receptor)	985	0.791	-0.0166	No
4	PIK3CG	PIK3CG Entrez, Source	phosphoinositide-3-kinase, catalytic, gamma polypeptide	1362	0.646	-0.0363	No
5	MYC	MYC Entrez, Source	v-myc myelocytomatosis viral oncogene homolog (avian)	1637	0.549	-0.0495	No
6	CPT1B	CPT1B Entrez, Source	carnitine palmitoyltransferase 1B (muscle)	4041	0.200	-0.2338	No
7	MRPL11	MRPL11 Entrez, Source	mitochondrial ribosomal protein L11	4097	0.195	-0.2352	No
8	SRA1	SRA1 Entrez, Source	steroid receptor RNA activator 1	4687	0.136	-0.2790	No
9	PRKAR2B	PRKAR2B Entrez, Source	protein kinase, cAMP-dependent, regulatory, type II, beta	4926	0.111	-0.2959	No
10	PTGS2	PTGS2 Entrez, Source	prostaglandin-endoperoxide synthase 2 (prostaglandin G/H synthase and cyclooxygenase)	4978	0.106	-0.2983	No
11	TNF	TNF Entrez, Source	tumor necrosis factor (TNF superfamily, member 2)	5084	0.097	-0.3050	No
12	APOA2	APOA2 Entrez, Source	apolipoprotein A-II	5361	0.069	-0.3255	No
13	FABP1	FABP1 Entrez, Source	fatty acid binding protein 1, liver	5865	0.020	-0.3644	No
14	EHHADH	EHHADH Entrez, Source	enoyl-Coenzyme A, hydratase/3-hydroxyacyl Coenzyme A dehydrogenase	6038	0.005	-0.3778	No
15	DUT	DUT Entrez, Source	dUTP pyrophosphatase	6105	-0.002	-0.3829	No
16	PRKAR1A	PRKAR1A Entrez,	protein kinase, cAMP-dependent, regulatory,	6916	-0.092	-0.4446	No

		Source	type I, alpha (tissue specific extinguisher 1)				
17	PRKAR1B	PRKAR1B Entrez, Source	protein kinase, cAMP-dependent, regulatory, type I, beta	7589	-0.185	-0.4942	No
18	APOA1	APOA1 Entrez, Source	apolipoprotein A-I	7601	-0.186	-0.4923	No
19	NR2F1	NR2F1 Entrez, Source	nuclear receptor subfamily 2, group F, member 1	9149	-0.517	-0.6052	No
20	CITED2	CITED2 Entrez, Source	Cbp/p300-interacting transactivator, with Glu/Asp-rich carboxy-terminal domain, 2	9558	-0.646	-0.6273	No
21	HSD17B4	HSD17B4 Entrez, Source	hydroxysteroid (17-beta) dehydrogenase 4	9694	-0.685	-0.6276	No
22	RB1	RB1 Entrez, Source	retinoblastoma 1 (including osteosarcoma)	9919	-0.755	-0.6338	No
23	PDGFA	PDGFA Entrez, Source	platelet-derived growth factor alpha polypeptide	10101	-0.816	-0.6358	No
24	MAPK1	MAPK1 Entrez, Source	mitogen-activated protein kinase 1	10270	-0.885	-0.6357	No
25	HSPA1A	HSPA1A Entrez, Source	heat shock 70kDa protein 1A	10448	-0.966	-0.6351	No
26	MAPK3	MAPK3 Entrez, Source	mitogen-activated protein kinase 3	10669	-1.047	-0.6366	Yes
27	EP300	EP300 Entrez, Source	E1A binding protein p300	10843	-1.126	-0.6333	Yes
28	NFKBIA	NFKBIA Entrez, Source	nuclear factor of kappa light polypeptide gene enhancer in B-cells inhibitor, alpha	10871	-1.138	-0.6185	Yes
29	SP1	SP1 Entrez, Source	Sp1 transcription factor	11093	-1.244	-0.6172	Yes
30	PRKACB	PRKACB Entrez, Source	protein kinase, cAMP-dependent, catalytic, beta	11106	-1.253	-0.5994	Yes
31	PPARGC1A	PPARGC1A Entrez, Source	peroxisome proliferative activated receptor, gamma, coactivator 1, alpha	11338	-1.376	-0.5969	Yes
32	STAT5B	STAT5B Entrez, Source	signal transducer and activator of transcription 5B	11445	-1.433	-0.5838	Yes

33	NR0B2	NR0B2 Entrez, Source	nuclear receptor subfamily 0, group B, member 2	11712	-1.617	-0.5805	Yes
34	PRKCA	PRKCA Entrez, Source	protein kinase C, alpha	11732	-1.637	-0.5576	Yes
35	STAT5A	STAT5A Entrez, Source	signal transducer and activator of transcription 5A	11901	-1.770	-0.5443	Yes
36	HSP90AA1	HSP90AA1 Entrez, Source	heat shock protein 90kDa alpha (cytosolic), class A member 1	12020	-1.877	-0.5255	Yes
37	DUSP1	DUSP1 Entrez, Source	dual specificity phosphatase 1	12276	-2.142	-0.5135	Yes
38	ACOX1	ACOX1 Entrez, Source	acyl-Coenzyme A oxidase 1, palmitoyl	12377	-2.288	-0.4872	Yes
39	PIK3CA	PIK3CA Entrez, Source	phosphoinositide-3-kinase, catalytic, alpha polypeptide	12385	-2.306	-0.4534	Yes
40	NCOA1	NCOA1 Entrez, Source	nuclear receptor coactivator 1	12411	-2.337	-0.4205	Yes
41	PRKAR2A	PRKAR2A Entrez, Source	protein kinase, cAMP-dependent, regulatory, type II, alpha	12439	-2.383	-0.3871	Yes
42	JUN	JUN Entrez, Source	jun oncogene	12478	-2.445	-0.3536	Yes
43	NRIP1	NRIP1 Entrez, Source	nuclear receptor interacting protein 1	12485	-2.466	-0.3174	Yes
44	RELA	RELA Entrez, Source	v-rel reticuloendotheliosis viral oncogene homolog A, nuclear factor of kappa light polypeptide gene enhancer in B-cells 3, p65 (avian)	12542	-2.568	-0.2835	Yes
45	PPARA	PPARA Entrez, Source	peroxisome proliferative activated receptor, alpha	12683	-2.903	-0.2511	Yes
46	PIK3R1	PIK3R1 Entrez, Source	phosphoinositide-3-kinase, regulatory subunit 1 (p85 alpha)	12717	-3.025	-0.2086	Yes
47	NCOR2	NCOR2 Entrez, Source	nuclear receptor co-repressor 2	12754	-3.204	-0.1637	Yes
48	NCOR1	NCOR1 Entrez, Source	nuclear receptor co-repressor 1	12838	-3.754	-0.1142	Yes

49	RXRA	RXRA Entrez, Source	retinoid X receptor, alpha	12841	-3.789	-0.0579	Yes
50	CREBBP	CREBBP Entrez, Source	CREB binding protein (Rubinstein-Taybi syndrome)	12861	-4.097	0.0016	Yes

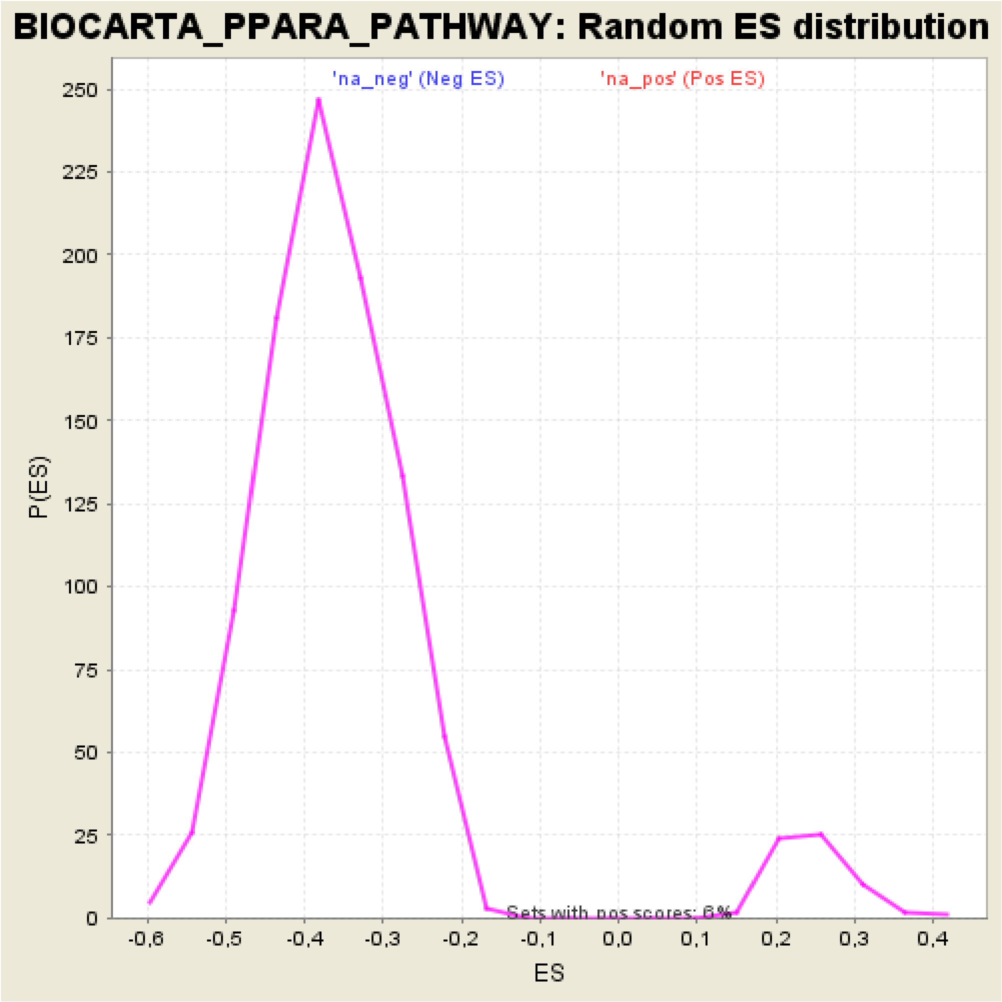

Fig 2: BIOCARTA_PPARA_PATHWAY: Random ES distribution
Gene set null distribution of ES for BIOCARTA_PPARA_PATHWAY
