# Supplementary material for: Atxn2 Knockout and CAG42-Knock-in Cerebellum Shows Similarly Dysregulated Expression in Calcium Homeostasis Pathway
Source: Cerebellum. 2016 Feb 11;16(1):68–81. doi: 10.1007/s12311-016-0762-4 (PMC5243904; doi:10.1007/s12311-016-0762-4)
Supplement: Supplementary file 4 — GSEA summary on BIOCARTA EGF pathway downregulation. (PDF 68 kb) [file 12311_2016_762_MOESM4_ESM.pdf]

Table: GSEA Results Summary

Dataset	GSEA_cbll_KO_collapsed
Phenotype	NoPhenotypeAvailable
Upregulated in class	na_neg
GeneSet	BIOCARTA_EGF_PATHWAY
Enrichment Score (ES)	-0.6985218
Normalized Enrichment Score (NES)	-1.7822206
Nominal p-value	0.0
FDR q-value	0.005530183
FWER p-Value	0.025

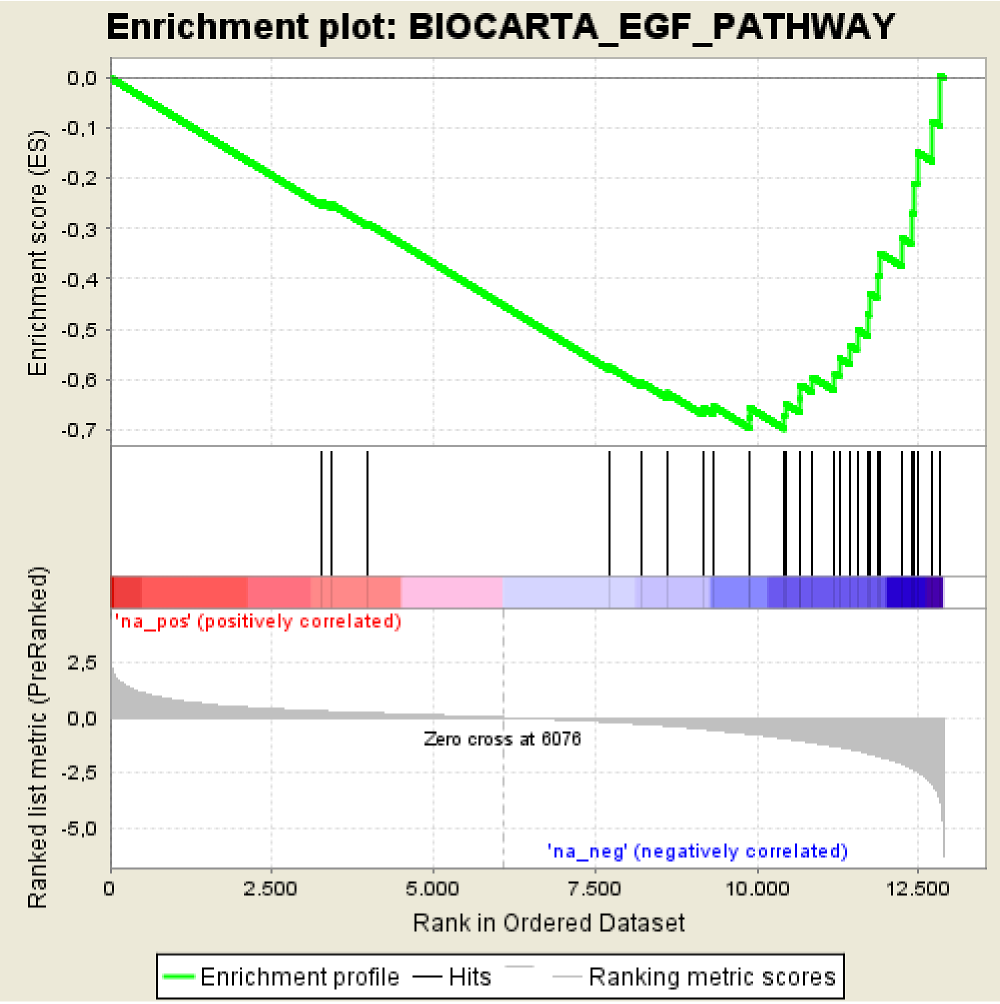

Fig 1: Enrichment plot: BIOCARTA_EGF_PATHWAY
Profile of the Running ES Score & Positions of GeneSet Members on the Rank Ordered List

Table: GSEA details [plain text format]

	PROBE	GENE	GENE_TITLE	RANK	RANK	RUNNING	CORE

		SYMBOL		IN GENE LIST	METRIC SCORE	ES	ENRICHMENT
1	EGF	EGF Entrez, Source	epidermal growth factor (beta-urogastrone)	3272	0.283	-0.2471	No
2	STAT4	STAT4 Entrez, Source	signal transducer and activator of transcription 4	3426	0.263	-0.2520	No
3	ELK1	ELK1 Entrez, Source	ELK1, member of ETS oncogene family	3987	0.206	-0.2901	No
4	SHC1	SHC1 Entrez, Source	SHC (Src homology 2 domain containing) transforming protein 1	7718	-0.204	-0.5749	No
5	FOS	FOS Entrez, Source	v-fos FBJ murine osteosarcoma viral oncogene homolog	8211	-0.290	-0.6054	No
6	MAP2K1	MAP2K1 Entrez, Source	mitogen-activated protein kinase kinase 1	8612	-0.372	-0.6267	No
7	RAF1	RAF1 Entrez, Source	v-raf-1 murine leukemia viral oncogene homolog 1	9176	-0.526	-0.6565	No
8	STAT1	STAT1 Entrez, Source	signal transducer and activator of transcription 1, 91kDa	9326	-0.573	-0.6529	No
9	SRF	SRF Entrez, Source	serum response factor (c-fos serum response element-binding transcription factor)	9879	-0.744	-0.6761	Yes
10	RASA1	RASA1 Entrez, Source	RAS p21 protein activator (GTPase activating protein) 1	9895	-0.748	-0.6574	Yes
11	GRB2	GRB2 Entrez, Source	growth factor receptor-bound protein 2	10424	-0.955	-0.6732	Yes
12	PLCG1	PLCG1 Entrez, Source	phospholipase C, gamma 1	10433	-0.958	-0.6484	Yes
13	MAPK8	MAPK8 Entrez, Source	mitogen-activated protein kinase 8	10666	-1.046	-0.6387	Yes
14	MAPK3	MAPK3 Entrez, Source	mitogen-activated protein kinase 3	10669	-1.047	-0.6110	Yes
15	STAT6	STAT6 Entrez, Source	signal transducer and activator of transcription 6, interleukin-4 induced	10859	-1.132	-0.5957	Yes
16	MAP3K1	MAP3K1 Entrez, Source	mitogen-activated protein kinase kinase kinase 1	11202	-1.309	-0.5876	Yes

17	CSNK2A1	CSNK2A1 Entrez, Source	casein kinase 2, alpha 1 polypeptide	11271	-1.344	-0.5572	Yes
18	STAT5B	STAT5B Entrez, Source	signal transducer and activator of transcription 5B	11445	-1.433	-0.5326	Yes
19	SOS1	SOS1 Entrez, Source	son of sevenless homolog 1 (Drosophila)	11546	-1.495	-0.5007	Yes
20	MAP2K4	MAP2K4 Entrez, Source	mitogen-activated protein kinase kinase 4	11718	-1.630	-0.4708	Yes
21	PRKCA	PRKCA Entrez, Source	protein kinase C, alpha	11732	-1.637	-0.4284	Yes
22	JAK1	JAK1 Entrez, Source	Janus kinase 1 (a protein tyrosine kinase)	11858	-1.729	-0.3922	Yes
23	STAT5A	STAT5A Entrez, Source	signal transducer and activator of transcription 5A	11901	-1.770	-0.3486	Yes
24	STAT2	STAT2 Entrez, Source	signal transducer and activator of transcription 2, 113kDa	12251	-2.111	-0.3197	Yes
25	PIK3CA	PIK3CA Entrez, Source	phosphoinositide-3-kinase, catalytic, alpha polypeptide	12385	-2.306	-0.2689	Yes
26	STAT3	STAT3 Entrez, Source	signal transducer and activator of transcription 3 (acute-phase response factor)	12436	-2.375	-0.2098	Yes
27	JUN	JUN Entrez, Source	jun oncogene	12478	-2.445	-0.1481	Yes
28	PIK3R1	PIK3R1 Entrez, Source	phosphoinositide-3-kinase, regulatory subunit 1 (p85 alpha)	12717	-3.025	-0.0863	Yes
29	EGFR	EGFR Entrez, Source	epidermal growth factor receptor (erythroblastic leukemia viral (v-erb-b) oncogene homolog, avian)	12835	-3.735	0.0037	Yes

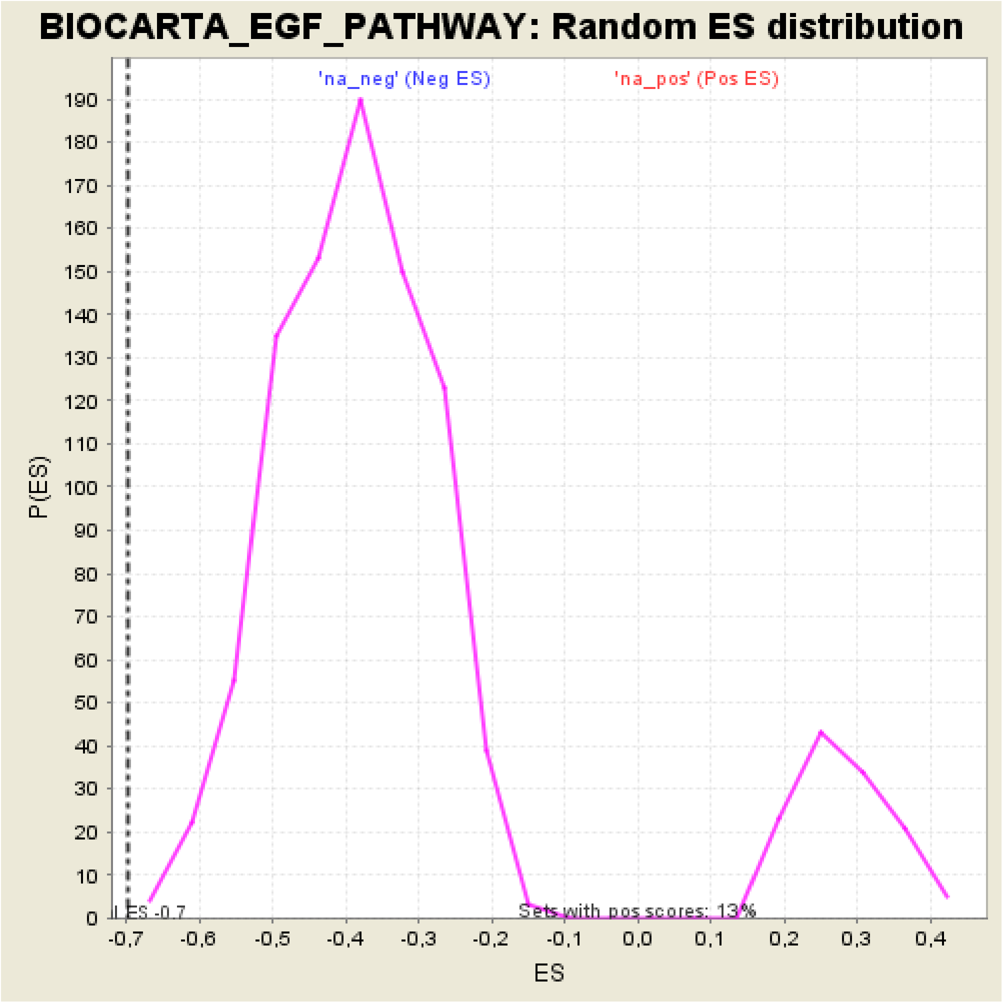

Fig 2: BIOCARTA_EGF_PATHWAY: Random ES distribution
Gene set null distribution of ES for BIOCARTA_EGF_PATHWAY
