# Supplementary material for: Atxn2 Knockout and CAG42-Knock-in Cerebellum Shows Similarly Dysregulated Expression in Calcium Homeostasis Pathway
Source: Cerebellum. 2016 Feb 11;16(1):68–81. doi: 10.1007/s12311-016-0762-4 (PMC5243904; doi:10.1007/s12311-016-0762-4)
Supplement: Supplementary file 5 — GSEA summary on BIOCARTA CREB pathway downregulation. (PDF 65 kb) [file 12311_2016_762_MOESM5_ESM.pdf]

Table: GSEA Results Summary

Dataset	GSEA_cbll_KO_collapsed
Phenotype	NoPhenotypeAvailable
Upregulated in class	na_neg
GeneSet	BIOCARTA_CREB_PATHWAY
Enrichment Score (ES)	-0.75261515
Normalized Enrichment Score (NES)	-1.8499733
Nominal p-value	0.0
FDR q-value	0.005227264
FWER p-Value	0.005

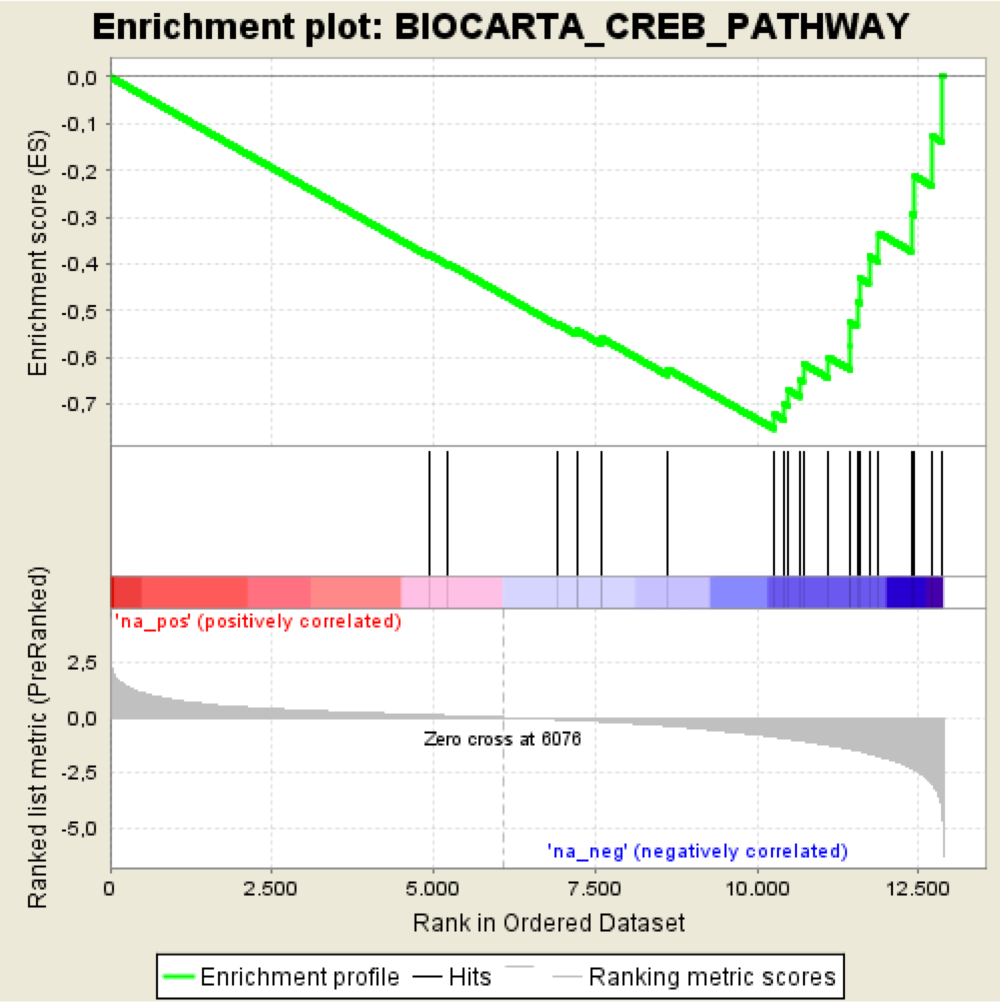

Fig 1: Enrichment plot: BIOCARTA_CREB_PATHWAY
Profile of the Running ES Score & Positions of GeneSet Members on the Rank Ordered List

Table: GSEA details [plain text format]

	PROBE	GENE	GENE_TITLE	RANK	RANK	RUNNING	CORE

		SYMBOL		IN GENE LIST	METRIC SCORE	ES	ENRICHMENT
1	PRKAR2B	PRKAR2B Entrez, Source	protein kinase, cAMP-dependent, regulatory, type II, beta	4926	0.111	-0.3792	No
2	RPS6KA5	RPS6KA5 Entrez, Source	ribosomal protein S6 kinase, 90kDa, polypeptide 5	5226	0.084	-0.3995	No
3	PRKAR1A	PRKAR1A Entrez, Source	protein kinase, cAMP-dependent, regulatory, type I, alpha (tissue specific extinguisher 1)	6916	-0.092	-0.5276	No
4	ADCY1	ADCY1 Entrez, Source	adenylate cyclase 1 (brain)	7223	-0.130	-0.5468	No
5	CAMK2A	CAMK2A Entrez, Source	calcium/calmodulin-dependent protein kinase (CaM kinase) II alpha	7224	-0.130	-0.5422	No
6	PRKAR1B	PRKAR1B Entrez, Source	protein kinase, cAMP-dependent, regulatory, type I, beta	7589	-0.185	-0.5641	No
7	CAMK2G	CAMK2G Entrez, Source	calcium/calmodulin-dependent protein kinase (CaM kinase) II gamma	7604	-0.188	-0.5586	No
8	CAMK2D	CAMK2D Entrez, Source	calcium/calmodulin-dependent protein kinase (CaM kinase) II delta	8619	-0.373	-0.6243	No
9	MAPK1	MAPK1 Entrez, Source	mitogen-activated protein kinase 1	10270	-0.885	-0.7215	Yes
10	GRB2	GRB2 Entrez, Source	growth factor receptor-bound protein 2	10424	-0.955	-0.6998	Yes
11	RPS6KA1	RPS6KA1 Entrez, Source	ribosomal protein S6 kinase, 90kDa, polypeptide 1	10485	-0.980	-0.6700	Yes
12	MAPK3	MAPK3 Entrez, Source	mitogen-activated protein kinase 3	10669	-1.047	-0.6475	Yes
13	MAPK14	MAPK14 Entrez, Source	mitogen-activated protein kinase 14	10711	-1.067	-0.6132	Yes
14	PRKACB	PRKACB Entrez, Source	protein kinase, cAMP-dependent, catalytic, beta	11106	-1.253	-0.5997	Yes
15	AKT1	AKT1 Entrez, Source	v-akt murine thymoma viral oncogene homolog 1	11438	-1.429	-0.5753	Yes
16	GNAS	GNAS Entrez,	GNAS complex locus	11441	-1.432	-0.5251	Yes

		Source					
17	SOS1	SOS1 Entrez, Source	son of sevenless homolog 1 (Drosophila)	11546	-1.495	-0.4806	Yes
18	RAC1	RAC1 Entrez, Source	ras-related C3 botulinum toxin substrate 1 (rho family, small GTP binding protein Rac1)	11587	-1.523	-0.4302	Yes
19	PRKCA	PRKCA Entrez, Source	protein kinase C, alpha	11732	-1.637	-0.3839	Yes
20	CREB1	CREB1 Entrez, Source	cAMP responsive element binding protein 1	11885	-1.752	-0.3341	Yes
21	PIK3CA	PIK3CA Entrez, Source	phosphoinositide-3-kinase, catalytic, alpha polypeptide	12385	-2.306	-0.2919	Yes
22	PRKAR2A	PRKAR2A Entrez, Source	protein kinase, cAMP-dependent, regulatory, type II, alpha	12439	-2.383	-0.2123	Yes
23	PIK3R1	PIK3R1 Entrez, Source	phosphoinositide-3-kinase, regulatory subunit 1 (p85 alpha)	12717	-3.025	-0.1275	Yes
24	CAMK2B	CAMK2B Entrez, Source	calcium/calmodulin-dependent protein kinase (CaM kinase) II beta	12855	-3.991	0.0021	Yes

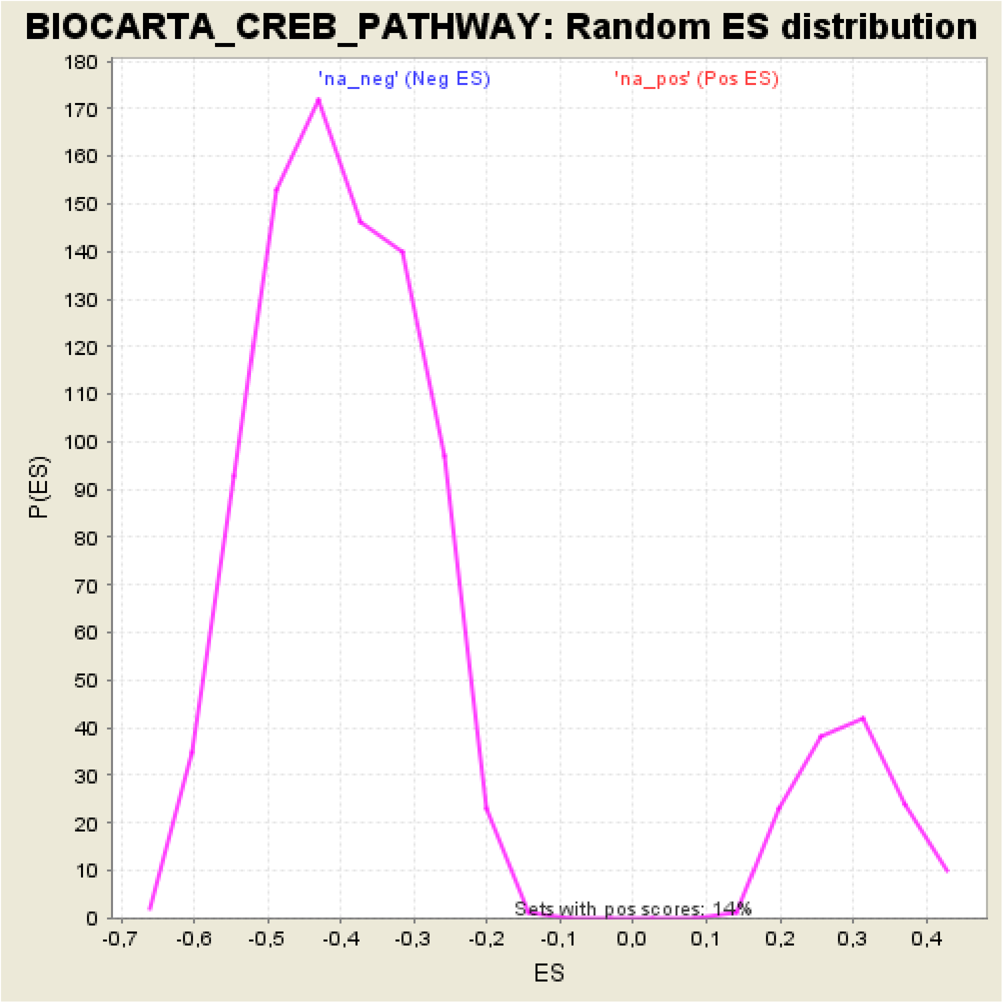

Fig 2: BIOCARTA_CREB_PATHWAY: Random ES distribution
Gene set null distribution of ES for BIOCARTA_CREB_PATHWAY
